# Benchmark and Parameter Sensitivity Analysis of Single-Cell RNA Sequencing Clustering Methods

**DOI:** 10.3389/fgene.2019.01253

**Published:** 2019-12-11

**Authors:** Monika Krzak, Yordan Raykov, Alexis Boukouvalas, Luisa Cutillo, Claudia Angelini

**Affiliations:** ^1^Institute for Applied Mathematics “Mauro Picone”, Naples, Italy; ^2^Department of Mathematics, Aston University, Birmingham, United Kingdom; ^3^Machine Learning Engineer Team, Prowler.io, Cambridge, United Kingdom; ^4^School of Mathematics, University of Leeds, Leeds, United Kingdom

**Keywords:** single-cell RNA-seq, clustering methods, benchmark, parameter sensitivity analysis, high-dimensional data analysis

## Abstract

Single-cell RNA-seq (scRNAseq) is a powerful tool to study heterogeneity of cells. Recently, several clustering based methods have been proposed to identify distinct cell populations. These methods are based on different statistical models and usually require to perform several additional steps, such as preprocessing or dimension reduction, before applying the clustering algorithm. Individual steps are often controlled by method-specific parameters, permitting the method to be used in different modes on the same datasets, depending on the user choices. The large number of possibilities that these methods provide can intimidate non-expert users, since the available choices are not always clearly documented. In addition, to date, no large studies have invistigated the role and the impact that these choices can have in different experimental contexts. This work aims to provide new insights into the advantages and drawbacks of scRNAseq clustering methods and describe the ranges of possibilities that are offered to users. In particular, we provide an extensive evaluation of several methods with respect to different modes of usage and parameter settings by applying them to real and simulated datasets that vary in terms of dimensionality, number of cell populations or levels of noise. Remarkably, the results presented here show that great variability in the performance of the models is strongly attributed to the choice of the user-specific parameter settings. We describe several tendencies in the performance attributed to their modes of usage and different types of datasets, and identify which methods are strongly affected by data dimensionality in terms of computational time. Finally, we highlight some open challenges in scRNAseq data clustering, such as those related to the identification of the number of clusters.

## Introduction

Single-cell RNA sequencing (scRNAseq) has emerged as an important technology that allows profiling gene expression at single-cell resolution, giving new insights into cellular development (Biase et al., 2014; Goolam et al., 2016), dynamics (Vuong et al., 2018; Farbehi et al., 2019), and cell composition (Darmanis et al., 2015; Zeisel et al., 2015; Segerstolpe et al., 2016). Although the scRNAseq analysis inherits many features from bulk RNA-seq approaches, the algorithms require constant adaptation due to the several types of challenges present in scRNAseq data (Kiselev et al., 2019). For example, current droplet-based technologies allow measuring hundreds of thousands of cells which greatly exceeds the number of samples typically handled by bulk RNA-seq protocols. The low amount of measured RNA transcripts per cell and stochastic nature of the genes expression can also introduce missing information about gene profiles (dropouts). The scRNAseq data specific noise and the increasing number of scRNAseq protocols differing in accuracy and scalability (Svensson et al., 2017; Svensson et al., 2018) make the systematic data analysis even more challenging.

Over the last few years, a number of computational algorithms have been proposed to analyze scRNAseq data, focusing on different aspects (Chen et al., 2019). In particular, a growing class of computational methods is being developed for identifying distinct cell populations (Andrews and Hemberg, 2018). These methods are based on various types of clustering techniques, which aim to divide cells into groups that share similar gene expression patterns. In this way, each group can be associated with a specific cell type or subtype on the basis of well-known markers, or novel cell subtypes can be identified. However, before applying the clustering algorithm, such methods often require to perform a series of mandatory or optional steps that include preprocessing, filtering or dimension reduction (Luecken and Theis, 2019). In several cases, such steps can be adapted by the user by choosing an appropriate set of parameters. Thus, methods turn to be very heterogeneous in the way they model data and perform the individual steps. Differences arise at each stage of the analysis and are not yet fully understood. For example, some algorithms work with raw count dataset (Zurauskiene and Yau, 2016; Lin et al., 2017; Sun et al., 2018), others require normalized gene expression values (Macosko et al., 2015; Ji and Ji, 2016; Senabouth et al., 2019) or can handle both formats (Yip et al., 2017; Qiu et al., 2017; Kiselev et al., 2017; Wang et al., 2017). Some of the tools do incorporate an additional method-specific preprocessing step in terms of filtering or normalization (Senabouth et al., 2019; Yip et al., 2017), to remove noise present in the data, other require such step to be done externally before the execution of the method (Julia et al., 2015). In addition to preprocessing, many methods often utilize dimension reduction techniques, such as Principal Component Analysis (PCA) or t-Distributed Stochastic Neighbor Embedding (tSNE), in order to reduce the high-dimensional space (expression of tens of thousands of genes) prior to clustering (Julia et al., 2015; Herman and Grün, 2018; Ren et al., 2019).

Another great difference is given by the specific clustering techniques implemented in each method. Some of the methods use partitioning algorithms (Kiselev et al., 2017; Wang et al., 2017) in order to infer distinct cell populations, others are based on hierarchical clustering (Senabouth et al., 2019; Lin et al., 2017), graph theory (Macosko et al., 2015) or density based-approach (Ester et al., 1996). There is also a growing class of model-based algorithms (Fraley and Raftery, 2002; Ji and Ji, 2016; Sun et al., 2018) which utilize probabilistic properties of a given model to account for distinct challenges present in the data. Moreover, some methods require the number of cell populations to be known in advance (Zurauskiene and Yau, 2016; Sun et al., 2018), while others estimate the optimal value with an external procedure or as part of the clustering inference (Macosko et al., 2015; Senabouth et al., 2019; Ren et al., 2019). The available methods also vary in terms of the programming language they have been implemented in (i.e. R, Matlab, Python), computational cost and other system requirements.

All of the mentioned variations across clustering pipelines affect the performance of the methods. Currently, there is a limited amount of studies that infer clustering performance and robustness under various data-driven scenarios (Freytag et al., 2018; Duò et al., 2018; Tian et al., 2019). The main purpose of existing studies is to investigate the performance of the methods limited to a selected parameter setting. Such limitation leads to a narrow view on the performance of the methods making it difficult to explore their full potential and identify the open challenges. For example, some algorithms provide multiple possibilities in the choice of parameters (Julia et al., 2015; Qiu et al., 2017; Herman and Grün, 2018; Ren et al., 2019) that can allow the user to adapt/modify the main method in each step. At the same time, the selection of parameter settings can be crucial in various data-driven conditions. The performance of the algorithms can also depend on the presence or absence of any preprocessing steps, either external or method-specific, carried out prior to clustering. Since both, parameter settings and data preprocessing can greatly affect the clustering result, we decided to investigate both aspects on the performance of the methods by carrying a comprehensive benchmark of the existing clustering methods and performing parameter sensitivity analysis.

For that purpose, we first described different modes of usage and parameter settings of 13 among the most widely used scRNAseq clustering methods implemented in R, then we applied them on a large set of real scRNAseq and simulated datasets. In order to fully understand the potential of each method, we tested them varying a wide range of available parameter settings which greatly expands the number of possible results. Through the analysis pipeline, we evaluated the performance of the methods with respect to several factors. First, we divided the real datasets into two groups, those that were expressed in the raw counts and those expressed on normalized fragments per kilobase of transcript per million mapped reads (FPKM) or reads per kilobase of transcript per million mapped reads (RPKM) counts. On the first group, we evaluated the performance of the methods on three data basic preprocessing types (not preprocessed counts, filtered counts, filtered and normalized counts). On the second group, we evaluated the performance of the methods depending on a various number of dimensions supplied to dimension reduction techniques prior to clustering. Synthetic datasets were used to prove the capacity of each method in handling varying dataset dimensions that can additionally be diverse in the number of simulated cell groups and the type of group balance. In the simulation, we also accessed the accuracy of the methods in recovering cell population structure in the presence of noise. The type of noise that we simulated were dropouts and overlapping cell populations which are key features of scRNAseq datasets. In all cases, we evaluated the performance of the methods in terms of i) Adjusted Rand Index (ARI) index, ii) accuracy of methods in estimating the correct number of clusters, iii) running time.

Overall, this work aims to provide new insights into the advantages and drawbacks of several scRNAseq clustering methods, by describing the ranges of possibilities that are offered to users and the impact that these choices can have on the final results. We also tried to identify some open challenges for future research that still need to be faced when doing the population inference.

## Materials and Methods

### Real Datasets

In order to evaluate the performance of the clustering methods considered in this study we used 17 real scRNAseq datasets popular in the literature and listed in **Table 1**. To prepare the gene expression matrix for clusterization, we followed the main instructions for data import and processing from the online repository https://hemberg-lab.github.io/scRNA.seq.datasets/.

**Table 1:** List of the real datasets used to perform the clustering evaluation.

Single cell dataset	Organism	Cells under study	Protocol	Accession
Baron2016_m	Mouse	Pancreas	inDrop	GSE84133
Klein2015	Mouse	Embryonic stem cells	inDrop	GSE65525
Zeisel2015	Mouse	Cerebral cortex	STRT/C1 UMI	GSE60361
Darmanis2015	Human	Brain	SMARTer	GSE67835
Deng2014_raw	Mouse	Preimplantation embryos	Smart-Seq	GSE45719
Goolam2016	Mouse	Early embryos	Smart-Seq2	E-MTAB-3321
Kolodziejczyk2015	Mouse	Stem cells	SMARTer	E-MTAB-2600
Li2017	Human	Colorectal tumors	SMARTer	GSE81861
Romanov2016	Mouse	Hypothalamus	Fluidigm C1	GSE74672
Tasic2016_raw	Mouse	Brain	SMARTer	GSE71585
Deng2014_rpkm	Mouse	Preimplantation embryos	Smart-Seq	GSE45719
Segerstolpe2016	Human	Pancreas	Smart-Seq2	E-MTAB-5061
Tasic2016_rpkm	Mouse	Brain	SMARTer	GSE71585
Xin2016	Human	Pancreas	SMARTer	GSE81608
Yan2013	Human	Preimplantation embryos	Tang	GSE36552
Biase2014	Mouse	Embryos	SMARTer	GSE57249
Treutlein2014	Mouse	Lung epithelial cells	SMARTer	GSE52583

Datasets (named by the author and date of publication) contain gene expression of cells from various organisms and tissues that have been processed by different experimental protocols. Protocols include 3’ or 5’ tag and droplet-based approaches (inDrop and STRT/C1 UMI), or full-length plate-based approaches, such as Smart-Seq, Smart-Seq2, SMARTer or Fluidigm C1. Tang protocol corresponds to mRNA-Seq assay described in (Tang et al., 2009). For more information about protocols see (Svensson et al., 2018).

The selected scRNAseq datasets vary in terms of organisms, tissues under study and experimental protocols. As illustrated in **Table 1**, some datasets were profiled using 3′ or 5′ tag and droplet-based approaches (such as inDrop), others using full-length plate-based approaches, such as Smart-Seq protocols. Moreover, depending on the used platform, each study investigates a different number of cells and data are subjected to a different proportion of dropouts. Depending on the protocol, count matrices were of different types (see **Table 2**) including Raw unique molecular identifier (UMI) counts (3 datasets), Raw read counts (7 datasets) and FPKM/RPKM counts (7 datasets). The raw counts (either UMI or read counts) consist of datasets with gene expression quantified in terms of the number of mapped reads (counts) and that have not been further processed, while FPKM or RPKM data are library size and gene length adjusted counts. Note that two datasets in **Table 2**, Deng2014 and Tasic2016, were of both types (raw read counts and FPKM/RPKM counts). Overall, the datasets cover various ranges of experimental complexity in terms of the number of sequenced cells (from tens to several thousands) and number of cell populations in the sample (with minimum of 3 and maximum of 18 number of cell populations). The cell populations (hidden groups to detect) can represent distinct cell types or cells at various time points of differentiation. Within this study, we will consider the cell population annotation (available from the corresponding datasets studies) as ground truth, although we are aware that there could be some errors in the annotations, since datasets could contain some rare cell subpopulations, that were not identified at the time of the study, or some misclassified cells.

**Table 2:** Brief description of the main features of each real dataset considered in this study.

Single cell dataset	Data type	Nr cells	Nr cell populations	Publication
Baron2016_m	Raw UMI counts	1886	13	Baron et al. (2016)
Klein2015	Raw UMI counts	2717	4	Klein et al. (2015)
Zeisel2015	Raw UMI counts	3005	9	Zeisel et al. (2015)
Darmanis2015	Raw read counts	466	9	Darmanis et al. (2015)
Deng2014_raw	Raw read counts	268	6	Deng et al. (2014)
Goolam2016	Raw read counts	124	4	Goolam et al. (2016)
Kolodziejczyk2015	Raw read counts	704	3	Kolodziejczyk et al. (2015)
Li2017	Raw read counts	561	9	Li et al. (2017)
Romanov2016	Raw read counts	2881	7	Romanov et al. (2016)
Tasic2016_raw	Raw read counts	1679	18	Tasic et al. (2016)
Deng2014_rpkm	RPKM	268	6	Deng et al. (2014)
Segerstolpe2016	RPKM	3514	15	Segerstolpe et al. (2016)
Tasic2016_rpkm	RPKM	1679	18	Tasic et al. (2016)
Xin2016	RPKM	1600	8	Xin et al. (2016)
Yan2013	RPKM	90	6	Yan et al. (2013)
Biase2014	FPKM	56	4	Biase et al. (2014)
Treutlein2014	FPKM	80	5	Treutlein et al. (2014)

Datasets can contain counts of 3 different types: Raw UMI counts, Raw read counts, and normalized FPKM/RPKM counts. Raw counts stands for the non-normalized counts that differ in terms of gene expression quantification method. FPKM/RPKM counts mean library size and gene length normalized counts. The number of reported cell populations is obtained from the annotation as described in the corresponding datasets publications.

### Simulated Datasets

**Figure 1:**
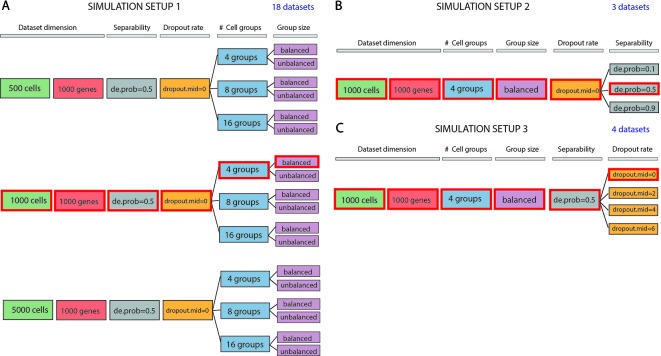
Data simulation scheme. **(A)** Simulation of 18 datasets using Setup 1. Simulated datasets are of various dimensions (number of cells), number of cell groups and proportion of cells within each group (balance or unbalance group sizes). **(B)** Simulation of 3 datasets using Setup 2. Simulated datasets vary in terms of separability between the groups (from poorly to well separable). This feature has been controlled by setting the de.prob parameter of Splatter simulation function to three values: 0.1, 0.5 and 0.9. **(C)** Simulation of 4 datasets using Setup 3. In this simulation setup, we used one dataset to create 3 others by placing an increasing number of zeros (controlled by dropout.mid parameter) on the count matrix. We highlighted by red color three identical datasets across all simulated setups. Each simulation setup has been repeated with 5 different values of the seed.

We evaluated methods performance also on synthetic datasets. The simulation study was performed using Splatter package (Zappia et al., 2017). Splatter allows simulating single-cell RNA sequencing count data with a varying number of cells and cell groups, with different degree of cluster separability and varying rate of dropouts. We designed three simulation setups that allowed us to investigate various aspects of the performance of the methods (see **Figure 1**). Each simulation setup has been repeated 5 times choosing 5 different values of the seed.

In the first simulation setup (**Figure 1A**), we focused on assessing both the scalability (the capacity of each method in handling datasets with an increasing number of cells) and the complexity of the dataset (the ability of each method when the number of true groups increases or when the balancing between each group is disrupted). For this purpose, we simulated counts using three different values for the number of cells: 500, 1000 and 5000; three values for the number of groups: 4, 8, 16 and two possibilities for the number of cells in each of the group: balanced and unbalanced group size. In each of the modes, we set the number of genes to 1000. Therefore, the resulting 18 simulated datasets represent different levels of data complexity and size for the clustering task.

In the second simulation setup (**Figure 1B**), we fixed dataset dimension (1000 cells, 1000 genes) as well as the cell groups (fixed to four groups balanced in sizes) and we investigated the performance of each method with respect to the group separability ranging from poorly to well-separated groups. In such setup, we varied the probability of a gene to be differentially expressed to 0.1, 0.5, and 0.9, to obtain 3 simulated datasets: expression probability close to 1 gives highly separable cell groups that should be less difficult to be detected by any clustering algorithm.

Finally, in the third simulation setup (**Figure 1C**), we investigated the performance of clustering methods in the presence of various rates of missing information. With the number of cells and genes the same as before (1000) and cell groups fixed to four, we varied the rate of zero counts by setting the midpoint parameter (drop.mid) for dropout logistic function to 0, 2, 4, and 6. In this way, we obtained 4 datasets with varying percentage of dropouts from 20% to 90%.

In each of the 5 runs of simulation, we have kept the synthetic datasets, highlighted in red in **Figures 1A–C** (i.e., those corresponding to 1000 cells, 1000 genes, 4 groups, size-balanced, de.prob = 0.5 and drop.mid = 0), identical across all three setups for easier direct comparison.

### Analysis Pipeline

**Figure 2:**
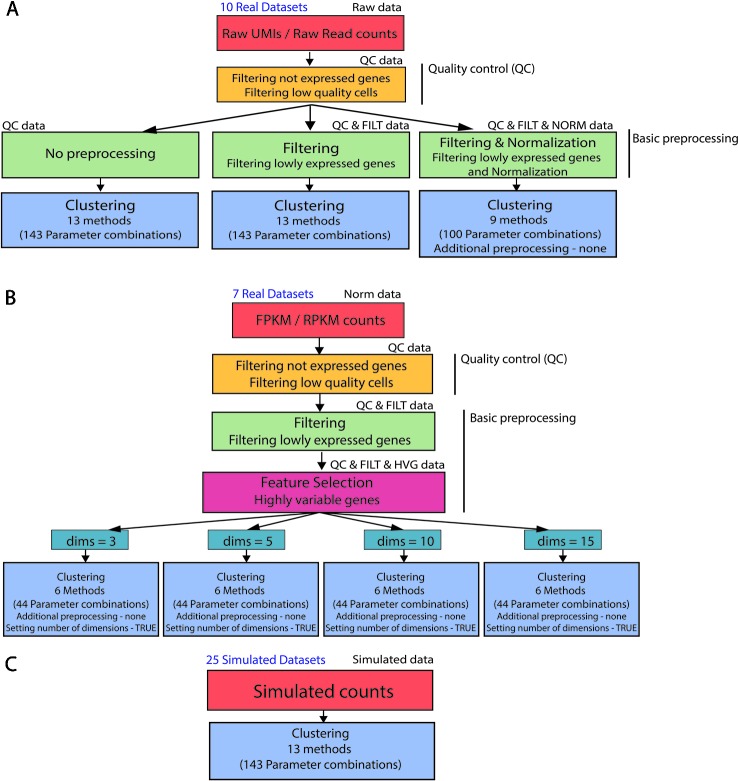
Clustering analysis pipeline. **(A) (B)** Real data analysis is divided into three steps: Quality control, basic preprocessing and clustering. **(C)** Clustering is directly applied to simulated datasets. Note that not all the parameter combinations have been applied to each dataset type. For filtered and normalized raw counts we excluded parameter combinations that use an additional method specific preprocessing. For FPKM/RPKM counts we used only those methods that do not allow for additional preprocessing (none) and provide option to set the number of reduced dimensions (TRUE).

In order to analyze real and simulated data, we used the procedure illustrated in **Figure 2**. First, all 17 real datasets (Raw UMI/Raw read counts and FPKM/RPKM counts) underwent the same quality control by filtering not expressed genes and low-quality cells (see **Figures 2A, B**) to remove potential issues from further analysis.

For the raw datasets, we considered three types of basic preprocessing before applying the specific clustering methods (**Figure 2A**). After the basic preprocessing, the clustering methods were applied with specific combinations of the parameters. Note that only a subset of methods (and combination of parameters) can be considered for filtered and normalized counts.

The FPKM/RPKM counts underwent a different basic preprocessing step (see **Figure 2B**) and were then directly clustered. To investigate the influence of the choice in the number of retained dimensions on methods performance, we considered only those methods and those combinations of parameters that allowed us to set the number of reduced dimensions.

In contrast to real data, simulated counts were directly used for clustering (see **Figure 2C**) where all methods and combination of parameters have been considered in the evaluation.

More details about data quality control, basic preprocessing of Raw and FPKM/RPKM counts, methods and parameter settings are described in the next sections.

### Quality Control of Real Datasets

All real datasets underwent an identical quality control step using the scater package (McCarthy et al., 2017). Firstly, we removed features with duplicated gene names and/or not expressed across all the cells as they do not include any useful information. Then, we performed quality control on the cells excluding those with the total number of expressed genes and the total sum of counts more than 3 median absolute deviations below the median across the genes [as suggested in scater documentation (McCarthy and Lun, 2019)]. Cells with the low amount of expressed genes and few counts are likely to be stressed or broken and thus should be removed from the analysis. The resulting dimensions of real datasets before and after quality control are given in **Supplementary Tables 1** and **2**.

### Basic Preprocessing of Real Datasets

After quality control, we applied a basic preprocessing step that mimics some of the most commonly used procedures typically applied before clustering scRNAseq data (McCarthy et al., 2017). In the case of Raw UMIs and Raw read counts, we considered three independent types of basic preprocessing: no preprocessing, filtering, filtering and normalization (see **Figure 2A**). Clearly, in the first case, no further operations were performed on the raw counts. In the second case, we used scater to remove lowly expressed genes that are genes with average expression count (adjusted by library size) equal to 0, where for the library size we mean total sum of the counts per cell. Note that this filtering step did not affect some of the datasets including Baron2016_m, Klein2015, Zeisel2015, and Romanov2016 (see **Supplementary Table 1**). In the third type, we first applied the filtering as described above, then we performed normalization. Both, Raw UMI counts and Raw read counts were normalized by scran package using deconvolution method. The deconvolution method normalizes data by cells-pooled size factors that account for dropout biases. More details about raw dataset dimensions before and after filtering are given in **Supplementary Table 1**. For illustrative purpose, **Supplementary Figure 1** reports one realization of the tSNE projections of the 10 raw datasets after quality control step that were colored by the corresponding cell group annotations. The inspection of the figure shows the heterogeneity of the datasets with respect to number of cells, number of cell groups and their separation.

In case of FPKM/RPKM counts, the basic preprocessing involved the same gene filtering as for the raw counts followed by high variable gene selection (HVG) (**Figure 2B**). To extract the most informative genes, we used Seurat package (Macosko et al., 2015) that defines most variable genes based on mean-variance dispersion. The dimensions of datasets before and after basic preprocessing are given in **Supplementary Table 2**. **Supplementary Figure 2** shows one realization of the tSNE projections (colored by the corresponding cell group annotations) of the 7 FPKM/RPKM datasets after quality control and basic preprocessing step.

### Compared Methods and Modes of Usage

In this study, we evaluated 13 different methods aimed to identify cell populations from scRNAseq data. **Table 3** lists the methods that we have considered. For the sake of code compatibility and transparency, we restricted our choice to the methods implemented in the R programming language. Some of the methods have multiple releases and versions. In this evaluation, we only tested the releases with versions reported in **Table 3**. For the sake of completeness, we stress that recently some of the methods listed in the table underwent to a major update which could have partially improved their performance.

**Table 3:** List of methods compared in the benchmark.

Method	Version	Class of clustering technique	Publication
ascend	v0.9.0	Hierarchical	Senabouth et al. (2019)
CIDR	v0.1.5	Hierarchical	Lin et al. (2017)
DIMMSC	v0.2.1	Model-based	Sun et al. (2018)
Linnorm	v2.6.1	Partitioning	Yip et al. (2017)
monocle3	v2.99.2	Multiple choices	Qiu et al. (2017)
pcaReduce	v1.0	Hierarchical	Zurauskiene and Yau (2016)
RaceID3	v0.1.3	Multiple choices	Herman and Grün (2018)
SC3	v1.10.1	Partitioning	Kiselev et al. (2017)
Seurat	v2.3.4	Graph-based	Macosko et al. (2015)
SIMLR	v1.8.1	Partitioning	Wang et al. (2017)
sincell	v1.14.1	Multiple choices	Julia et al. (2015)
sscClust	v0.1.0	Multiple choices	Ren et al. (2019)
TSCAN	v1.20.0	Model-based	Ji and Ji (2016)

Versions of the R packages (methods) compared in this study. Methods are based on various clustering techniques that can be categorized based on the cluster-model. Multiple choices indicate that the method allows to cluster cells with more than one clustering technique.

Most of the methods (i.e., all except DIMMSC and pcaReduce) considered in this study can be applied by setting different parameter combinations, thus providing potentially different results. Such combinations of parameters allow the user to tune different modes of usage, such as including or not an additional preprocessing step, including or not a dimension reduction procedure, using different criteria for choosing the suitable data dimension, applying different clustering algorithms within the same method, setting or estimating the number of clusters. **Table 4** shows a detailed series of parameters that the user can choose with possible parameter choices. Each row defines valid parameter settings for the specific method. Within the same row, the total number of combinations is given by the product of each possibility (the last column of **Table 4** summarizes the number of combinations). If the method has been reported more than once in the table (i.e., Linnorm and sscClust), it means that some of the parameters worked only with a subset of the settings (i.e., not in a full combinatorial way). By considering all possible combinations, we obtained 143 potential different modes of usage of the 13 tested methods.

**Table 4:** Valid configurations in the parameter settings for each method.

Method	Additional preprocessing	Dimension reduction	Setting number of dimensions	Clustering technique	Number of clusters	Combinations
ascend	method specific	internal	TRUE/internal	fixed	estimate	2
CIDR	none	internal	TRUE/internal	fixed	set/estimate	4
DIMMSC	none	none	FALSE	fixed	set	1
Linnorm	none/method specific	tSNE/PCA	TRUE/internal	fixed	set/estimate	16
Linnorm	none/method specific	none	FALSE	hclust	set	2
monocle3	none/method specific	tSNE/UMAP	TRUE/internal	densityPeak/louvain	estimate	16
pcaReduce	none	internal	internal	fixed	set	1
RaceID3	method specific	PCA	TRUE/internal	k-medioids/k-means/hclust	set/estimate	12
SC3	none/method specific	internal	internal	fixed	set/estimate	4
Seurat	method specific	PCA/ICA	TRUE/internal	fixed	estimate	4
SIMLR	none/method specific	internal	TRUE/internal	fixed	set/estimate	8
sincell	none	PCA/ICA/tSNE/classical-MDS/nonmetric-MDS	TRUE/internal	max.distance/percent/knn/k-mediods/ward.D	estimate	50
sscClust	none	iCor	internal	k-means/ADPclust/hclust	set/estimate	6
sscClust	none	iCor	internal	SNN	estimate	1
sscClust	none	PCA	TRUE/internal	k-means/ADPclust/hclust	set/estimate	12
sscClust	none	PCA	TRUE/internal	SNN	estimate	2
TSCAN	method specific	internal	internal	fixed	set/estimate	2

We reported a set of parameters that users can tune in the method such as the additional preprocessing, the dimension reduction strategy, the number of dimensions, the clusterring technique and the number of clusters to obtain. In particular, for the key additional processing: none – no additional preprocessing is applied, method specific – an additional preprocessing is applied prior clustering (filtering and/or normalization); for dimension reduction: internal – an internal dimension reduction is applied, none – the method works in the original domain, PCA, tSNE, ICA, iCor or others listed by names – the user can choose a specific method to reduce the dimensionality; for number of dimensions: TRUE or FALSE – method allows or doesn’t allow for setting number of reduced dimensions, internal – method use an internal value for the number of dimensions; for clustering technique: fixed –method uses only one clustering technique, otherwise the user can choose among few options that are listed by name; for number of clusters: set or estimate – method allows to set or estimate number of clusters.

As shown in **Table 4**, eight methods (corresponding to 43 parameter combinations) might incorporate an additional preprocessing step (herein, denoted method specific), five methods do not have any specific step (herein denoted none). Out of the 8 methods that include the additional preprocessing step, four methods allow the user to decide it to apply or not (both settings available). Methods differ also in the dimension reduction step either by providing only an internal procedure to reduce dimensions (six methods, herein denoted internal) or allowing for multiple choices for this purpose (five methods, herein denoted with the name of the specific procedures the user can choose, PCA, tSNE, ICA, etc). Note that two methods, DIMMSC and Linnorm have to or can, respectively, work directly in the high-dimensional space (setting herein denoted with none) and one method RaceID3 uses PCA dimension reduction which has been not considered as an internal technique (for more details see methods description in Supplementary Materials). Within all 12 methods that incorporate the dimension reduction step, an internal procedure can be used for selecting the number of reduced dimensions (herein denoted internal). Nine algorithms (63 combinations) also allows to manually set the number of dimensions (herein denoted with TRUE). Those with both options give to the user the possibility of either choosing the dimension or using the internal procedure. In this regard, the setting FALSE is related to methods that do not perform dimension reduction.

Methods can be also customized by the clustering techniques they apply. Some of them are based on a fixed clustering technique (herein denoted fixed), others propose multiple choices in this step (herein denoted with the name of the specific technique the user can choose, k-means, hclust, etc). The group of methods with multiple clustering options include: monocle3 that offers two types of clustering techniques, RaceID3 that utilizes two partitioning algorithms and a hierarchical clustering algorithm, sincell and sscClust which provide more clustering options. Depending on the clustering technique, methods either require to set the number of clusters by the user (36 combinations, herein denoted set) or provide an internal functionality to estimate it (107 combinations, herein denoted estimate). For more details about specifications, see methods descriptions in **Supplementary Materials**.

Finally, we stress that all 13 methods (with all 143 combinations of parameters) can be applied to non-preprocessed or filtered Raw counts as well as simulated datasets (see **Figure 2**). To avoid performing method-specific normalization on already normalized data, only methods for which the additional preprocessing step can be set to none were used on filtered and normalized Raw counts (i.e., 9 methods with 100 combinations of parameters) (**Figure 2A**) or FPKM/RPKM counts. In addition, according to **Figure 2**, when using normalized FPKM/RPKM counts, we reduced the number of methods and parameter combinations to those which perform dimension reduction step before clustering, and allow setting number of reduced dimensions in that step. In this way, we used a subset of 6 methods and 44 combinations of parameters to be applied on FPKM/RPKM counts (**Figure 2B**).

### Evaluation Metrics

To quantify the agreement between the partition obtained from the considered method and the true partition, we used a well-known and widely used measure, the Adjusted Rand Index (ARI), implemented in the R package mclust (Scrucca et al., 2016). The values of the ARI range can be negative if the agreement of the partitions is worse then the agreement expected by chance, or between 0 and 1 for clustering better than chance. The exact formulation of the ARI index can be found in (Lawrence and Phipps, 1985).

To evaluate the accuracy of methods in estimating the correct number of clusters, we used symmetric log-modulus transformation defined as follows:

(1) L(x)=sign(x)∗log10(|x|+1)

where *x* is the difference between the estimated number of clusters and the true number of cell populations in a given dataset. The positive values of log-modulus transformation mean that the number of estimated clusters was higher than the number of true cell populations. Negative values indicate that methods underestimate the number of clusters whereas zero values denote the equality between the number of estimated clusters and the number of true cell populations.

To identify significant differences in methods performance (ARI Index) when applied after different basic preprocessing types, we used hypothesis testing procedures implemented in stats R package (Hollander and Wolfe, 1973). The Kruskal-Wallis rank sum test was used to assess the difference in methods performance as we vary the basic preprocessing (among QC, QC & FILT, QC & FILT & NORM). The Wilcoxon signed-rank test was used to infer the differences in accuracy with respect to two data basic preprocessing types (QC, QC & FILT). In each context, we computed the Benjamini-Hochberg adjusted p-values (Benjamini and Hochberg, 1995) to correct for multiple comparisons.

Finally, to measure the computational time required by each method to complete its task, we used *Sys.time* function from R that allows reporting time when the method starts and finishes the script. The difference between those time points constituted the computational time of the method in running dataset analysis. Note that computational times have been reported in the unit of minutes followed by *log*(*t*+1) transformation, where *t* is the running time in minutes, and include all the steps that the method needs to cluster a dataset (except data basic preprocessing) together with the loading of the required packages and package dependencies.

### Implementation

This clustering benchmark study was implemented in the R programming language and scripts necessary for the reproducibility were deposited at the time of publication on the GitHub page: https://github.com/mkrzak/Benchmarking_Clustering_Methods_scRNAseq. The repository stores codes for data import, processing, and analysis as well as the information about system requirements and packages to be installed. When performing the analysis, additional HTML reports are produced with a detailed description of data analysis steps. Note that apart from the required methods, the analysis scripts call for other R packages used in plotting and managing R objects. The scripts have been tested on R version 3.5.1 and machine with specifications—Intel Core i7, 4.00 GHz × 8 and 24 GB RAM which are the minimum system requirements for the analysis.

Moreover, for the sake of completeness and to ensure the reproducibility of our study, we deposited the real and simulated datasets on the following GitHub pages: https://github.com/DataStorageForReproducibility/Real_data_for_benchmark_reproducibility and https://github.com/DataStorageForReproducibility/Simulated_data_for_benchmark_reproducibility. Both directories include. RData files as SingleCellExperiment class objects that store the count matrices and the corresponding cell group annotations.

In the clustering benchmark, we set the seed for generating pseudo-random numbers globally and applied it to the execution of any method in order to assure the stability of the solutions and reproducibility of the results. Note that, since the scRNAseq R packages we evaluated are often under continuous development, other version of the methods (R packages) than those reported in **Table 3**, might output slightly different results.

## Results

Results are organized as follows. We first illustrate the performance of the evaluated methods on the 10 raw datasets, then on the 7 normalized FPKM/RPKM counts. Finally, we finish the summary of the main findings obtained on the simulated datasets in the 3 setups described in **Figure 1**.

Within this paper, methods/parameter combinations are referred as string obtained as a concatenation of keys separated by underscores. The concatenation takes the name of the method, the type of additional preprocessing, the dimension reduction technique, the setting of the number of dimensions, the clustering technique and the number of clusters. Each of these keys can take the values reported in **Table 4**.

### Methods Performance on Raw UMI and Raw Read Counts

As mentioned, we independently applied all 13 methods (corresponding to 143 parameter combinations) to the 10 raw counts datasets after using two basic preprocessing types (QC, QC & FILT). Then, we applied only 9 methods (corresponding to a subset of 100 parameter combinations) to the same datasets after applying quality control, filtering and normalization (see the scheme illustrated in **Figure 2**). In the latter case, the 9 methods are those that allow the user to choose none as additional preprocessing to avoid renormalization of already normalized counts (see **Table 4**). To compare the methods across the basic preprocessing procedures, we first show the results corresponding to the combinations that were applied to all three basic preprocessing procedures, then the remaining methods/combinations applied only to QC and QC & FILT data.

Note that some of the methods/parameters combinations failed to cluster some datasets (such cases are marked in grey in **Supplementary Figures 3**and **4**) due to the errors occurred during their execution. The most frequent error messages were reported in **Supplementary Table 3**, for Data type = “Raw counts”. In particular, SIMLR, DIMMSC and Linnorm encountered failures in a limited number of cases, therefore we did not consider such datasets in the evaluation of the methods. By contrast, sincell (when ICA was chosen for dimension reduction) reported a significant number of failures, therefore we did not consider such combinations of parameters in the evaluation of sincell. Note that this will limit the overall number of parameter combinations from 143 to 133 (90 combinations applied after all three types of basic preprocessing, 43 applied to QC and QC & FILT data, only).

#### Overall Accuracy

**Figure 3:**
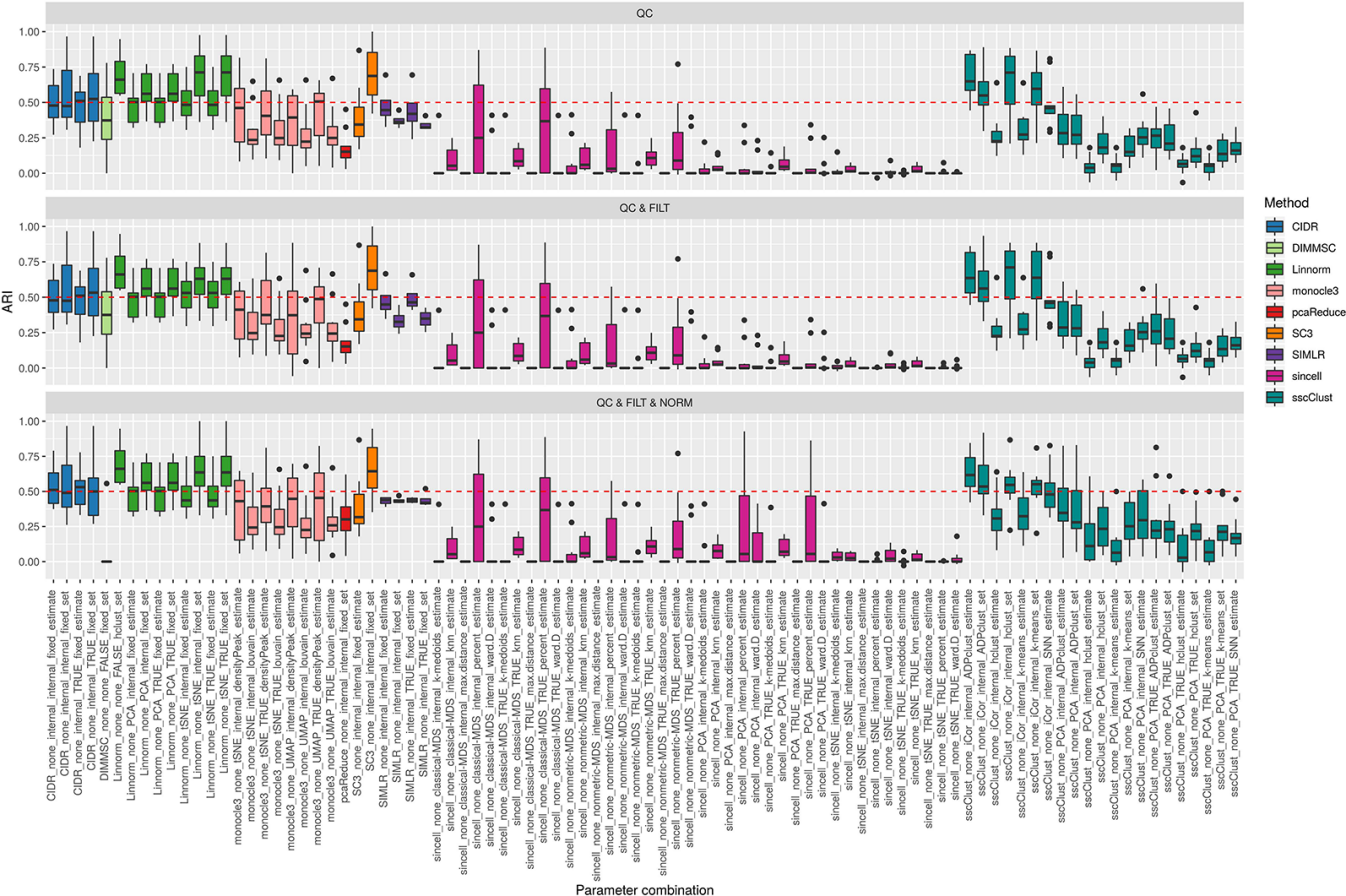
Overall accuracy of methods applied to Raw counts. ARI accuracy for 9 methods with 90 parameter combinations out of 100, independently applied to the 10 raw datasets after the three basic preprocessing types (QC, QC & FILT, QC & FILT & NORM). Box colors distinguish the different methods, although applied with different parameter combinations. Superimposed as reference, a red dashed line at ARI = 0.5.

**Figure 4:**
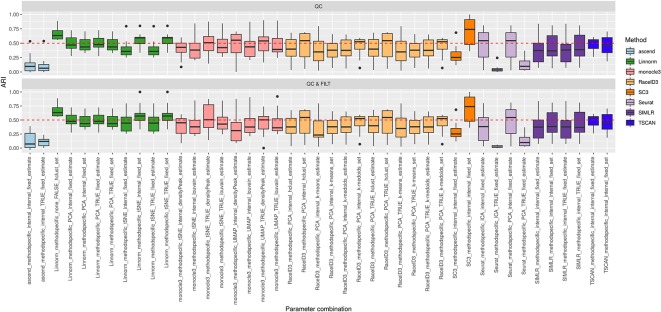
Overall accuracy of methods applied to Raw counts. ARI accuracy for remaining 8 methods with 43 parameter combinations, independently applied to the 10 raw datasets after two basic preprocessing types (QC, QC & FILT). Box colors distinguish the different methods, although applied with different parameter combinations. Superimposed as reference, a red dashed line at ARI = 0.5.

**Figure 3** shows the performance of the 9 methods (90 parameter combinations out of 100) in terms of ARI evaluated across all 10 raw datasets and organized with respect to the type of basic preprocessing. Analogously, **Figure 4** shows the same results corresponding to the remaining 8 methods (43 parameters combinations) independently applied after two basic preprocessing types. To evaluate the overall accuracy, we first inspected the results regardless of the type of basic preprocessing.

From **Figures 3** and **4**, we can observe that, most of the methods/parameter combinations report a great variability in their performance across the different datasets which proves no all-time winner across the entire set of cases we have analyzed. Some of the methods still performed relatively well (i.e., with most of the results above ARI = 0.5) regardless the preprocessing type. This group includes CIDR, Linnorm (with some combinations of parameters), SC3 (when set is chosen in number of clusters), some combinations of sscClust (i.e., when iCor is used for dimension reduction) and TSCAN. On the other hand, few other methods were reporting very poor performance. For example, one of the poorest performance was observed in sincell (with many parameter combinations), ascend, DIMMSC, pcaReduce and Seurat (only when non-internal is chosen for the number of reduced dimensions). Although sincell performed generally poor, the method also showed good performance for few datasets (see, the results over individual datasets showed in **Supplementary Figure 3**).

The analysis of **Figures 3** and **4** also shows that the performance of some methods strongly depends on the particular choice of the parameter settings, i.e. sscClust, Linnorm or Seurat being those whose performance strongly rely on that option. We found such result partially ignored in previous studies, therefore we will investigate it in more detail in *Effect of Parameters Settings on Accuracy*.

#### Accuracy in Estimating the Number of Clusters

In order to evaluate the accuracy of a method in estimating the correct number of populations, we used log-modulus transformation in Eq. 1, and we limited the analysis to the 107 methods/parameter combinations that allow setting the option estimate for choosing the number of clusters (see **Table 4**).

**Figure 5:**
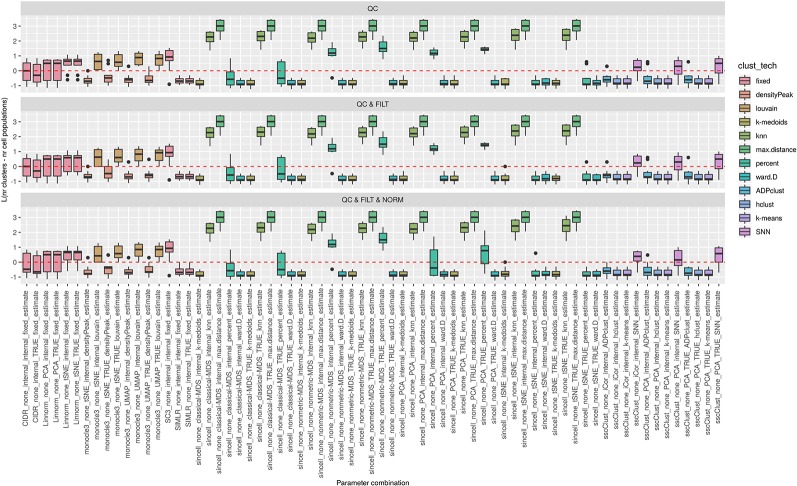
Estimation of the number of clusters for methods applied to Raw counts. Boxplots of *L* in Eq. 1 for the subset of methods (i.e., 69 parameter combinations) that allows to estimate the number of clusters (and with none preprocessing). Superimposed as a reference, a red dashed line at L = 0. Parameter combinations with difference below or above 0 resulted into under or overestimation of the number of clusters, respectively.

**Figure 6:**
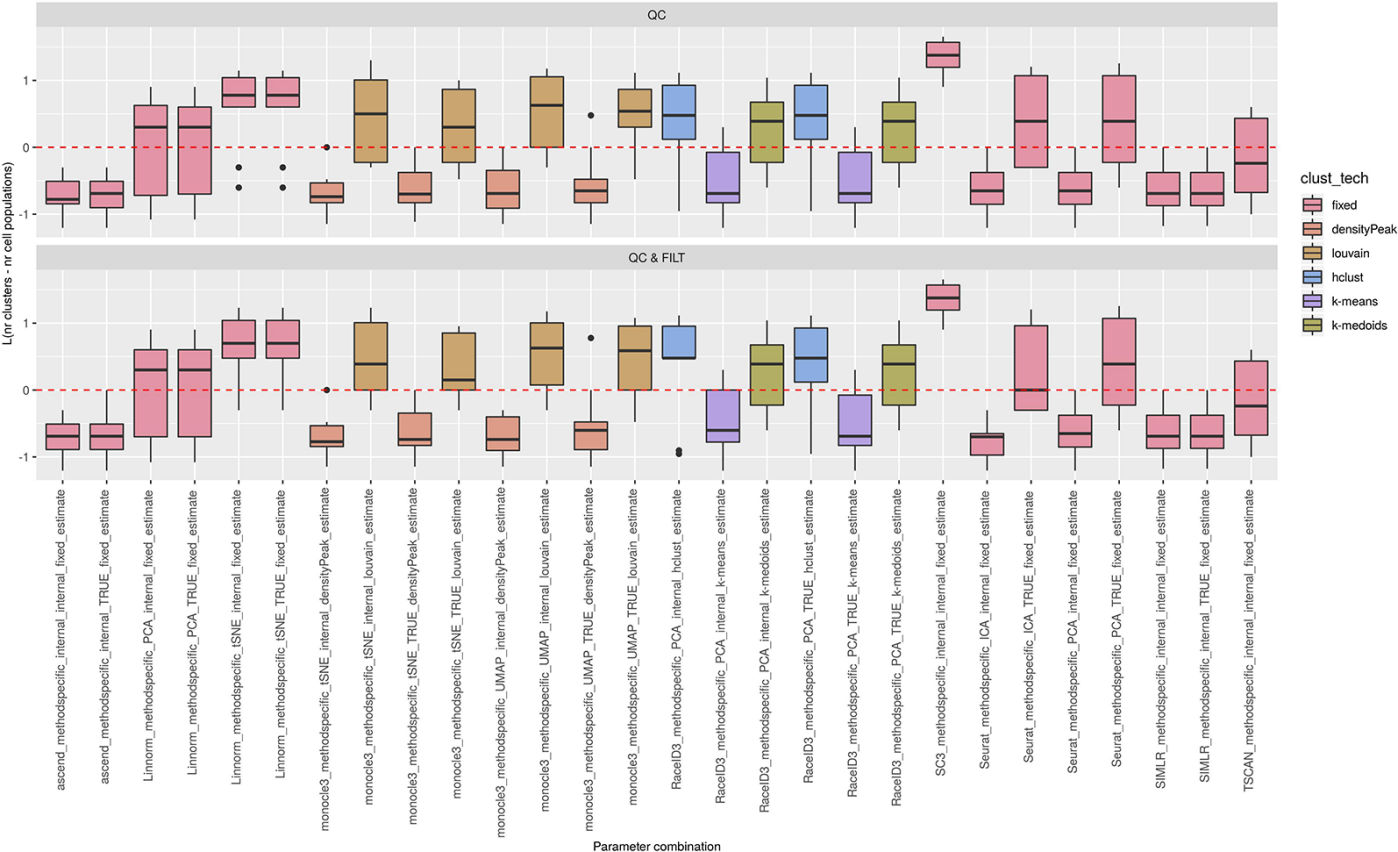
Estimation of the number of clusters for methods applied to Raw counts. Boxplots of *L* in Eq. 1 for the subset of methods for the remaining methods (28 parameter combinations) with method specific preprocessing that allows to estimate number of clusters. Superimposed as reference, a red dashed line at L = 0. Parameter combinations with difference below or above 0 resulted into under or overestimation of the number of clusters, respectively.

**Figures 5** and **6** show the results, respectively for the 69 methods/parameters combinations applied after all three types of preprocessing procedures (i.e., we excluded 10 combination of sincell that reported frequent failtures), and for the remaining 28 methods/parameter combinations applied after two basic preprocessing steps.

We observed that most of the methods/parameter combinations either under or overestimated the number of clusters often in a systematic way. In particular, boxes below and above the dashed lines represent parameter combinations which under or overestimated the number of clusters. There are also methods, such as CIDR, some combinations of Linnorm, RaceID3 and TSCAN, that often provide less biased estimates. We also observed that the estimates strongly depend on the specific clustering technique used, as for monocle3, sincell and RaceID3 method, or dimension reduction applied, as for Linnorm. The group of methods that underestimated number of clusters includes monocle3 (when densityPeak is used for clustering), SIMLR method, sincell (with k-medoids and ward.D chosen as clustering techniques), all combinations of sscClust except SNN and RaceID3 (when k-means was applied). A special case of overestimating the number of clusters method was observed with sincell where a large number of cluster was often returned. For example, sincell used with max.distance technique always returned a number of clusters equal to the number of cells in the dataset whereas in combination with knn it also returned a very large number of clusters.

#### Effect of Data Basic Preprocessing on Accuracy

We found that most of the methods performed similarly when changing the preprocessing procedures (see **Figures 3** and **4**), although **Supplementary Figures 3** and **4** showed that some of them (i.e., Linnorm, monocle3, sincell and sscClust) present slight variability in the performance when data underwent to different preprocessing. However, Kruskal-Wallis rank sum test did not identify any significant difference in the performance of the methods with respect to the three types of basic preprocessing (QC, QC & FILT, QC & FILT & NORM) and Wilcoxon signed-rank test did not identify any significant difference associated to the two types of basic preprocessing (QC, QC & FILT).

#### Effect of Parameter Settings on Accuracy

As mentioned above, **Figures 3** and **4** clearly shows that the performance of several methods depends much more on the choice of parameters than on the type of basic preprocessing.

**Figure 7:**
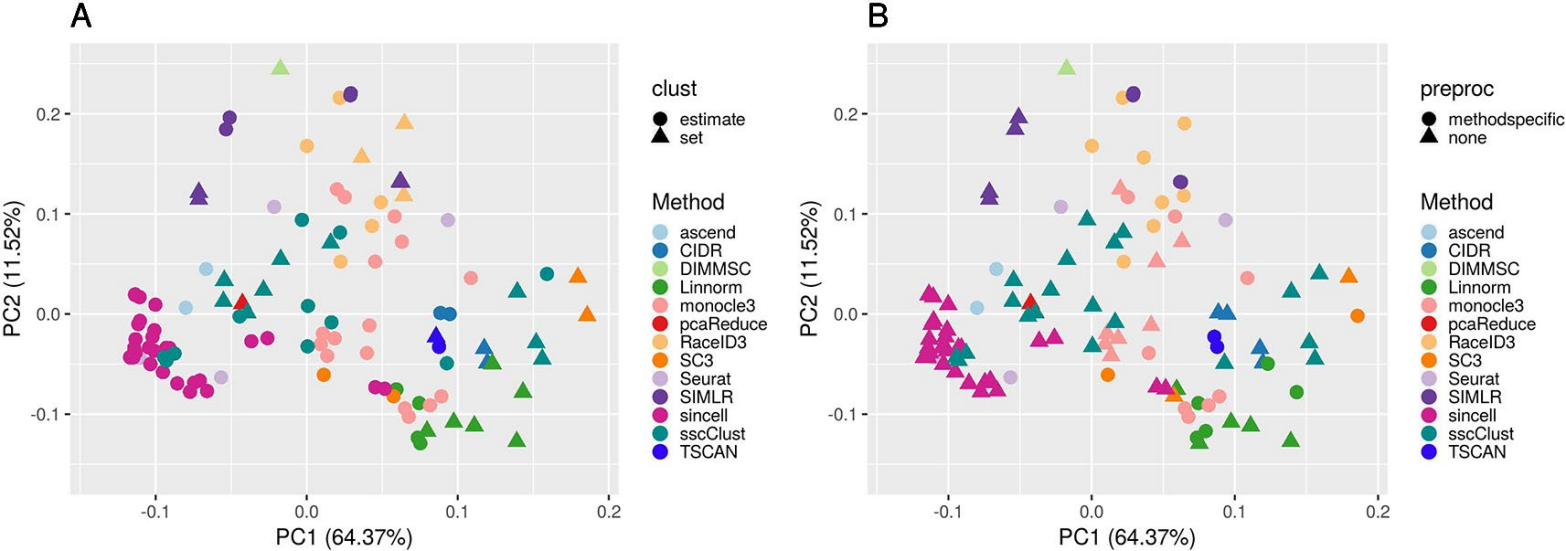
PCA plots of methods applied to QC & FILT Raw counts. Two identical PCA projections based on the performance measured in ARI of 13 methods with 133 parameter combinations out of 143, applied to 10 quality controlled and filtered (QC & FILT) raw datasets. Parameter combinations were colored by the method and shaped by the parameter options: **(A)** way of selecting number of clusters (clust), **(B)** additional preprocesing (preproc).

To better investigate this, we computed the PCA of the ARI matrix obtained using the 133 methods/combination as variables and the 10 raw datasets as samples. **Figure 7** shows the results when the clustering methods were applied to QC & FILT preprocessed data (the figures after the other preprocessing types are very similar, not shown for brevity). Each point depicted in the PCA space represents a particular methods/parameter combination. Therefore, points that are close in the PCA space have similar performance across the 10 datasets. From **Supplementary Figure 5** we can see that the first component is strongly positively correlated with the performance, therefore methods located on the right side of the figure tends to have better performance than those located on the left side, while the second component is not significantly correlated with the ARI. Each panel of **Figure 7** represents the same PCA projection colored by the methods and shaped by one of the parameters of interest. The effect of parameters changes in the performance of a given method is represented through the spread of the points in the same color. Note that DIMMSC and pcaReduce have only one valid parameter combination thus we do not discuss them in this section, although they are depicted in the figure.

Overall, **Figure 7** confirms the poor performance of sincell and the good performance of SC3, CIDR, TSCAN, and some combinations of Linnorm, as well as the strong impact of parameters setting for many methods (i.e., sscClust, Linnorm, Seurat, SIMLR).

In particular, the analysis of **Figure 7A** shows that the performance strongly depends on whether the number of clusters is estimated or not. Not surprisingly, when using the true number of clusters (parameter set) the performance is better for most of the methods compared to when estimating it (parameter estimate). However, there are few methods that report good overall performance also when the number of clusters is estimated (see for example, CIDR, monocole3 and sscClust).

In the same spirit, **Figure 7B** illustrates the effect of an additional preprocessing (that can be either method specific or none) on the methods performance. The figure does not indicate any global difference, but still pointed-out some methods specific variability (i.e. SIMLR showed significantly improved accuracy after such step).

We also superimposed other features, such as dimension reduction or clustering techniques (not shown for brevity). Since such parameters can assume multiple values, the figures do not allow to identify any suggestion that works well for all methods. However, such analysis allowed us to recognize i.e., sscClust with iCor and Seurat with internal number of reduced dimensions, as one of the good performing combinations.

#### Computational Time

We compared run times of the methods across all 10 raw datasets in order to assess their scalability and identify potential issues related to a specific dataset.

**Figure 8:**
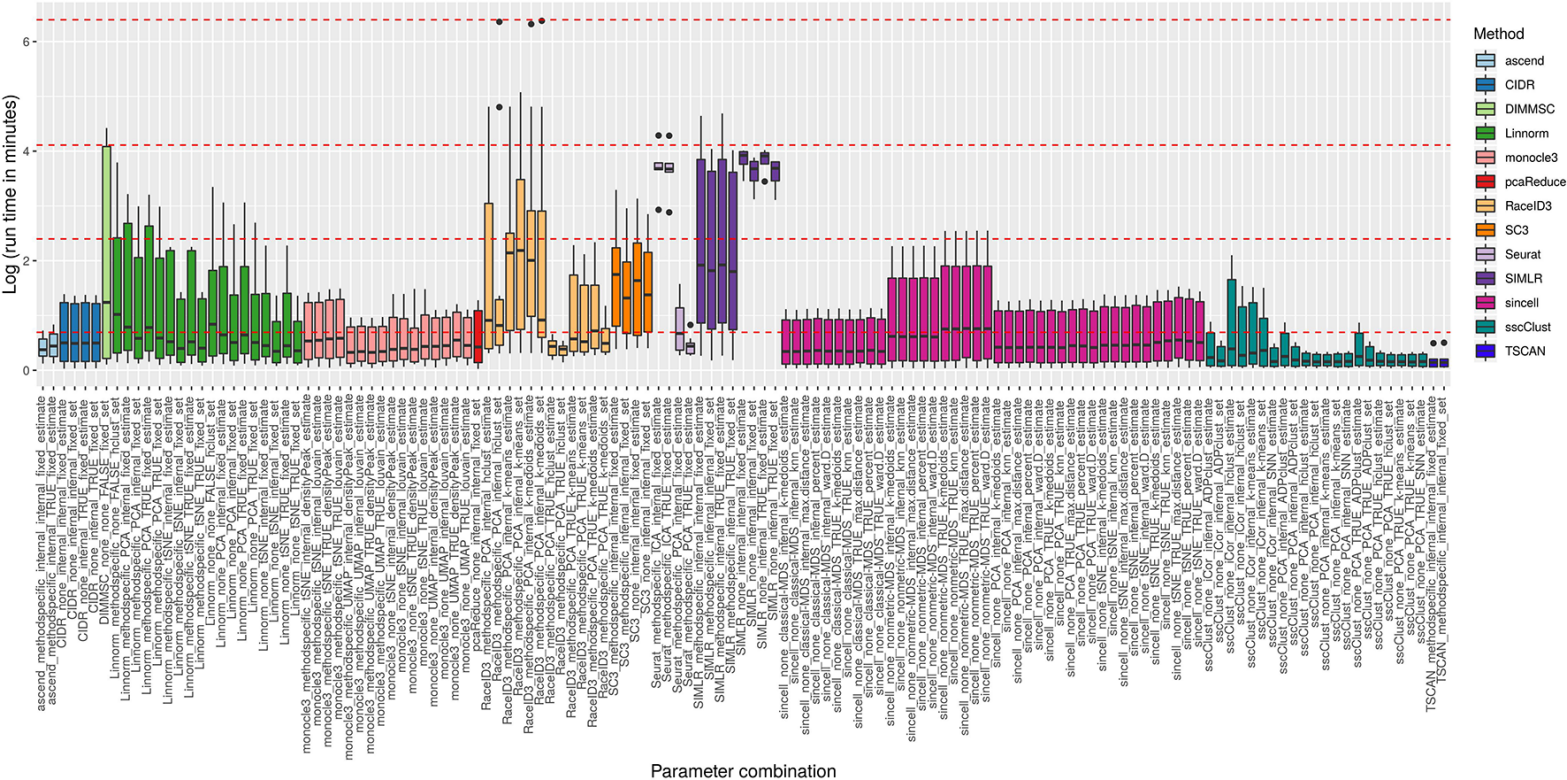
Computational time of methods applied to QC & FILT Raw counts. Log of run times in minutes of 13 methods with 133 parameter combinations applied to QC & FILT preprocessed raw datasets. We superimposed as reference red dashed lines at log of 1 min, 10 min, 1 h and 10 h.

**Figure 8** reports execution time in minutes on a log plus one scale for the methods applied to QC & FILT preprocessed datasets. As a reference, we superimposed on the figure dashed lines at 1, 10, 60 min, and 10 hours. Overall, computational times varied from a few seconds to tens of minutes or till several hours (at least for some datasets). We distinguish methods that were consistently fast (showing good scalability), methods requiring longer but still reasonable run time with increased data size (showing limited scalability) and methods requiring significant execution time at least in some cases (showing either poor scalability or problems related to the analysis of a specific dataset). ascend, CIDR, monocle3, pcaReduce, RaceID3 (with non-internal number of dimensions), Seurat (with PCA dimension reduction), sincell, sscClust and TSCAN were among the fastest and across the analyzed datasets. Therefore, they were assigned to the first group (with average run time below 2 minutes and maximum time of about 10 minutes). Linnorm and SC3 were assigned to the second group (with average run time about 5 minutes and maximum time between 20 minutes and an hour). Other methods such as DIMMSC, SIMLR, RaceID3 (with internal number of dimensions), Seurat (with ICA dimension reduction) were among the longest, therefore assigned to the third group (with average run time between 10 minutes and about two hours and maximum time between an hour and more than 10 hours). In the worst case, RaceID3 took about 12 hours before completing the clustering task.

### Methods Performance on FPKM/RPKM Counts

We used 7 FPKM/RPKM datasets to evaluate the performance of the methods/parameter combinations considered in this study with respect to the number of reduced dimensions when using different dimension reduction techniques. Since FPKM/RPKM datasets consist of already normalized counts we limited the study to those methods/parameter combinations that do not use “method specific” as additional preprocessing and that also allow setting the number of reduced dimensions. In total, we tested 44 methods/parameter combinations on each of the four dimensions: 3, 5, 10, and 15.

As in the previous case, we note that some of the methods/parameters combinations failed to cluster some of the datasets (see grey boxes in **Supplementary Figure 6**) due to technical errors reported in **Supplementary Table 3**, for Data type = FPKM/RPKM counts. Note that three of the methods, Linnorm, monocle3 and sincell encountered a significant number of failures with the same error message when used with more than 3 dimensions. We did not consider such cases in further evaluation limiting the overall number of combinations from 44 to 33.

#### Overall Accuracy

**Supplementary Figure 7** shows the performance of all 33 methods/parameter combinations applied to FPKM/RPKM datasets. Regardless of the number of dimensions, we can observe variability in the accuracy of the methods similar to what was reported for the raw counts. Most of the methods that were reporting good or poor accuracy on raw counts show similar good/poor performance also on the FPKM/RPKM datasets (as we could have predicted from the results obtained on the QC & FILT & NORM raw datasets). For example, CIDR and sscClust (with some of the parameter combinations) are among the better-performing methods, whereas sincell with most of the combinations reports poor accuracy (although not in all cases). Additionally, we can also confirm that the performance of some methods depends on the choice of parameter settings.

We also observed a general tendency of the methods to perform poorly on datasets with a high number of cells (more than 1600) (see **Supplementary Figure 6**). Although this relationship was not clearly visible on the raw counts, it could be expected as a consequence of a larger complexity in the data not fully explained by the number of selected features and not fully captured using low dimensional projections.

Finally, we did not observe any systematic differences in the accuracy with respect to the number of reduced dimensions (see **Supplementary Figures 6** and **7**). Some of the methods are either robust to the varying number of dimensions or they do not show any clear preference when using one or another setting. This suggests that data complexity cannot be easily explained by a certain parameter and the performance of the methods are often data specific.

#### Accuracy in Estimating Number of Clusters

**Supplementary Figure 8** shows the estimated number of clusters compared with the true one (as computed using Eq. 1) for all methods/combinations that allow the users to estimate such value. We observed a similar tendency in the estimates reported for raw counts. For example, monocle3 (with densityPeak clustering), SIMLR, sincell (with k-medoids and ward.D techniques) or sscClust (all except SNN) tend to underestimate the number of clusters whereas the rest of the combinations of sincell clearly overestimate that value. Moreover, CIDR often provides a less bias estimates that result in a better accuracy (alike on the raw counts).

#### Computational Time

**Supplementary Figure 9** reports the running time evaluated for all methods/parameter combinations (for dimension = 3). First, we observe that, since FPKM/RPKM counts underwent to a feature selection step, that greatly reduced the data dimension in terms of the number of genes (see **Supplementary Table 2**), we have a consequent reduction of the running time for most of the methods. Indeed, we can see that most of the methods ran below a minute (CIDR, monocle3 and sscClust) or in few minutes (some cases of sincell). SIMLR was the longest method and took up to one hour. Our study also shows that the number of reduced dimensions (3, 5, 10, or 15) was not so relevant in terms of computational time (data not shown).

Finally, note that some of the combinations evaluated on the raw counts, such as RaceID3 or Seurat, were not considered in the FPKM/RPKM evaluation as they do not allow to set none in the additional preprocessing.

### Methods Performance on Simulated Datasets

Synthetic datasets were used to test the performance of all 143 methods/parameter combinations. We followed three simulation setups in order to simulate the counts (see **Figure 1**) and we repeated the simulation 5 times, each with a different selection of the random seed. Simulation setups mimic different characteristics of scRNAseq datasets i.e. in terms of dimensionality, group structure or levels of noise. In theory, all simulated datasets constitute a different level of complexity for the clustering task.

The methods/parameter combinations that failed across all runs can be seen in the **Supplementary Figure 10** and the respective error messages have been reported the **Supplementary Table 3**.

In the next sections, we will describe the performance of the methods according to the three simulation setups. Note that the overall performance of the methods on the synthetic datasets is much higher than in the real data. This can be related to the fact that simulation models may not always reflect all types of noise present in the real case and thus the clustering task can be less challenging. Despite that, synthetic datasets allowed us to confirm some of the previously identified trends and to recognize the potential limits of the methods.

#### Performance on the Simulation Setup 1

**Figure 9:**
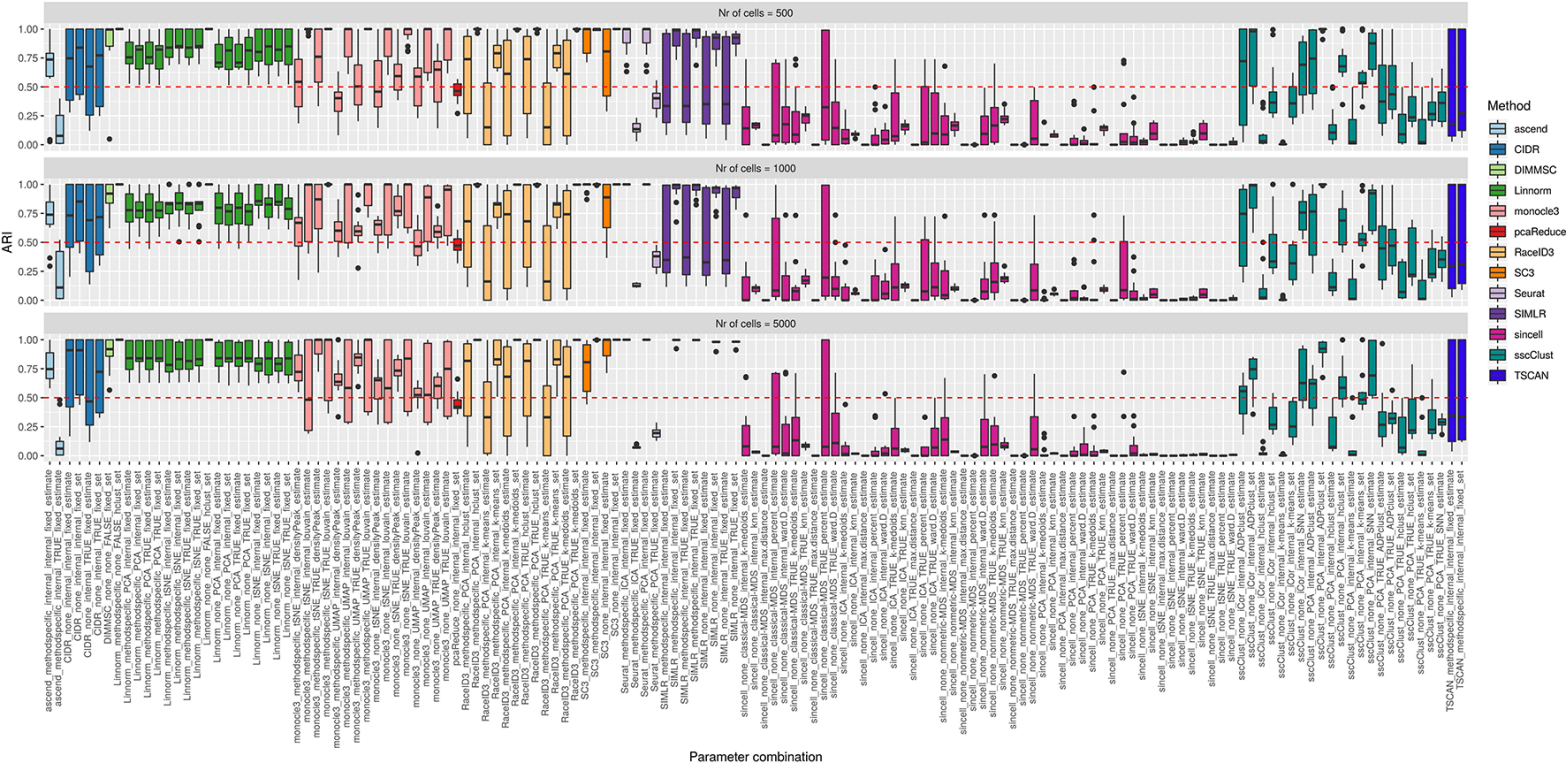
Overall accuracy of the methods on simulated datasets from Setup1 with balanced group sizes. Performance of 143 parameter combinations on Setup 1 simulated data. Selected results are across all runs.

Simulation Setup 1 has been used to access the performance of the methods depending on three factors: the number of cells present in the dataset, the number of cell groups and their balance in size. **Figure 9** and **Supplementary Figure 11** show the accuracy of the methods for balanced and unbalanced group design, respectively. **Supplementary Figures 12–14** give more details about balanced group design and correspond to the performance on datasets with 4, 8, and 16 number of cell groups, respectively.

By looking across the **Supplementary Figures 12–14** we can observe high variability of the methods/parameter combinations across different numbers of simulated cell groups. Less variability was attributed to the runs (see boxplots within **Supplementary Figures 12–14**). Balanced or unbalanced group design slightly affected the performance for most of the methods however with no clear direction (see **Figure 9** and **Supplementary Figure 11**).

On the synthetic datasets, the well performing methods included CIDR, Linnorm, SC3 and some combinations of sscClust (see **Figure 9** and **Supplementary Figure 11**), same as for the real datasets. Similarly, we could confirm the poor performance of methods such as Seurat with an imposed number of dimensions or sincell with tSNE dimension reduction. Additionally, on the synthetic data we observed high accuracy of the DIMMSC method, Seurat with internal number of dimensions and some combinations of monocle3, RaceID3 and SIMLR.

We did not observe a large loss in methods performance when the number of cells increased from 500 to 5000 (**Figure 9** and **Supplementary Figure 11**). The only clear exception was SIMLR with several combinations that include cluster number estimation (denoted estimate) which failed on datasets with 5000 number of cells (see the error messages in **Supplementary Table 3**). We observed that many methods were affected by the growing number of simulated cell groups (from 4 to 16 cell groups). In particular, see the methods: CIDR, DIMMSC, Linnorm, SC3, SIMLR, sincell, sscClust, and TSCAN across **Supplementary Figures 12–14**. pcaReduce worked similarly across all three factors (number of cells, number of cell groups, group balance) (see **Figure 9** and **Supplementary Figure 11**). Seurat accuracy, same for the real datasets, strongly dependent on the number of reduced dimensions (denoted as TRUE/internal).

#### Performance on the Simulation Setup 2

In the simulation Setup 2, we varied the separability between the cell groups from lowly to highly separable. Lowly separable groups mean that some of the simulated populations could overlap in space being the most challenging to detect. Separability was controlled by de.prob parameter in the Splatter simulation function.

**Figure 10:**
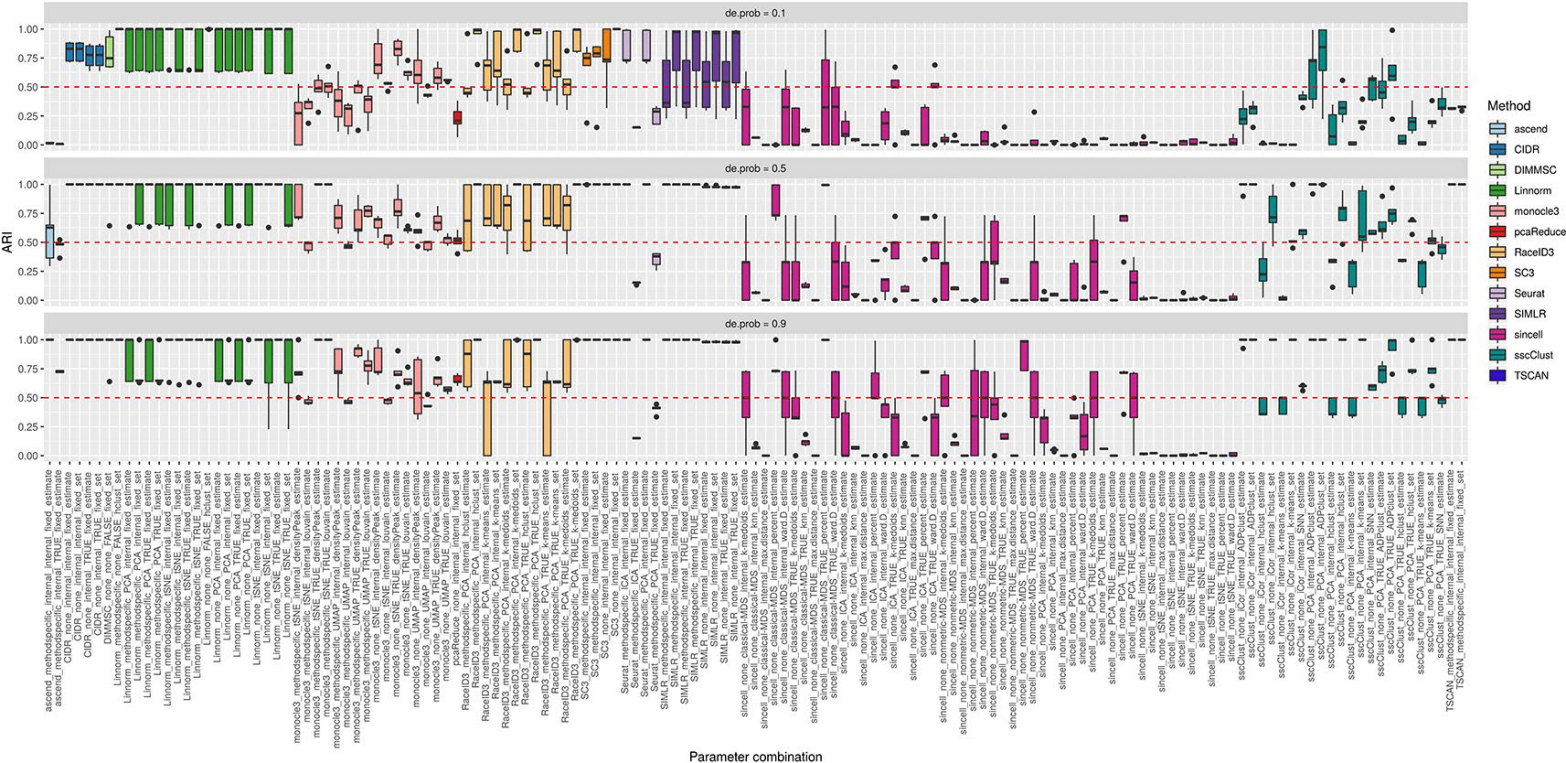
Overall accuracy of the methods on simulated datasets from Setup 2. Performance of 143 parameter combinations on simulated data. Selected results are across all runs.

**Figure 10** shows that some of the methods as CIDR, DIMMSC, SC3, TSCAN, Seurat (with imposed number of dimensions), SIMLR (with estimated number of clusters) and many combinations of sincell behaved similarly and their performance was mostly affected on the datasets with the lowest separability between the cell groups. However, their accuracy was still high meaning in most of the cases ARI above 0.5. The methods that performed well across all the separability modes were some combinations of Linnorm or monocle3, Seurat with internal number of dimensions and SIMLR with set number of clusters. All those methods/parameter combinations provided high accuracy with ARI close to 1.

#### Performance on the Simulation Setup 3

The third simulation setup was used to access the accuracy of the methods with respect to an increasing number of zero counts placed in the dataset. We simulated percentage of dropouts varying from 20% to 90% by manipulating dropout.mid parameter in the Splatter simulation function.

**Figure 11:**
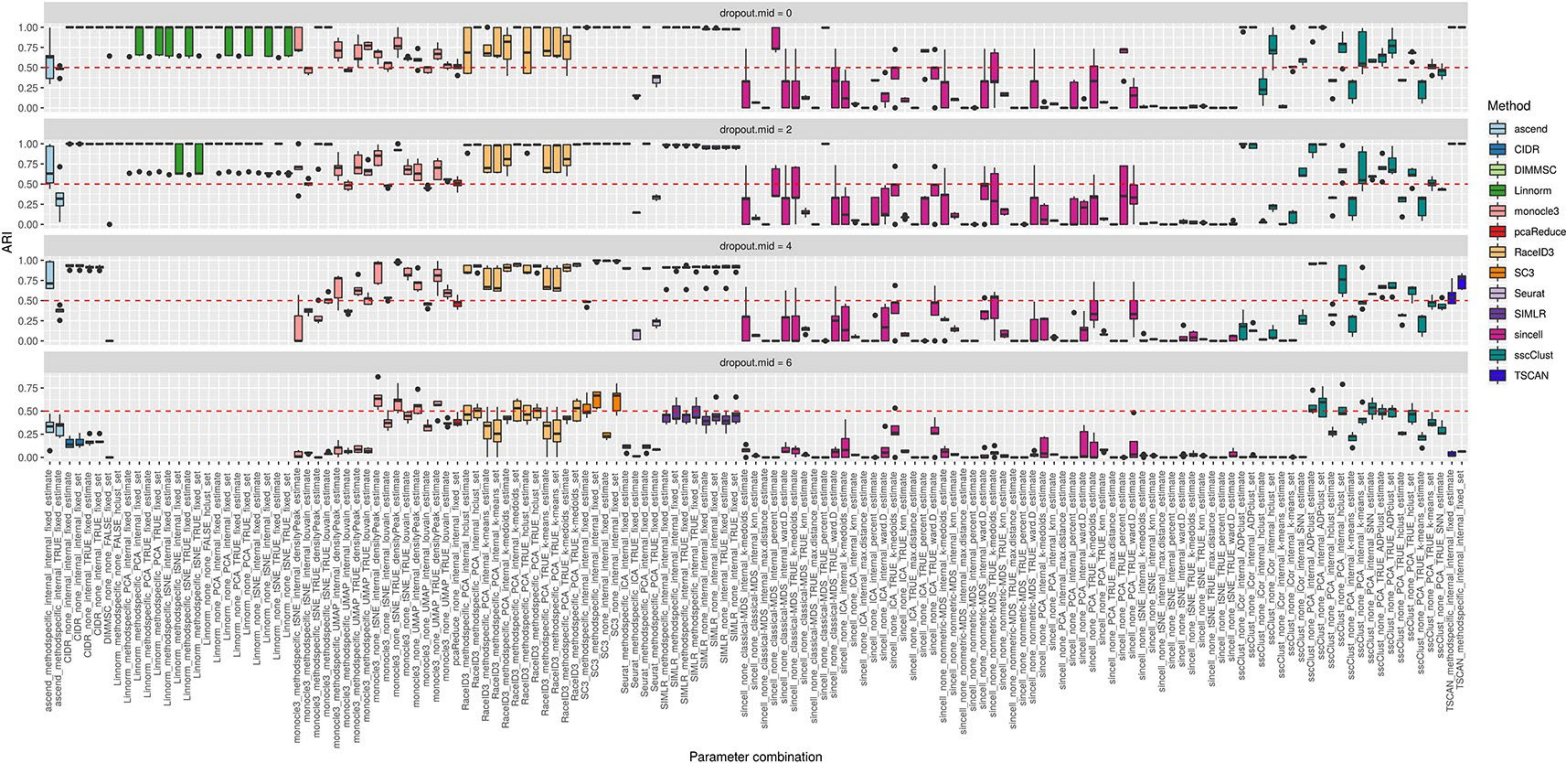
Overall accuracy of the methods on simulated datasets from Setup 3. Performance of 143 parameter combinations on simulated data. Selected results are across all runs.

Overall, we noticed that most of the methods had low accuracy on the datasets with highest magnitude of missing values (dropout.mid = 6) (see **Figure 11**). Although this is an expected result, some of the methods/parameter combinations still performed well in this case (see i.e. monocle3, SC3 and sscClust). Interestingly, monocle3 and sscClust method performed poorly only in particular parameter combinations on the highest dropout rate. For the monocle3 method the bad performing combinations included additional method specific preprocessing and for the sscClust—iCor dimension reduction. Beyond that, some of the methods appeared to be affected by the increasing percentage of zeros, as CIDR, DIMMSC, Linnorm, Seurat, SIMLR, and TSCAN. In particular, Linnorm experienced technical errors across all the simulated datasets with the highest two modes of dropouts (denoted as dropout.mid = 4 and dropout.mid = 6). Many combinations of sincell performed poorly, notably those that use tSNE as dimensionality reduction. Seurat depended highly on the number of dimensions used (either TRUE or internal) and pcaReduce seemed to work moderate across all four ranges of dropouts.

#### Computational Time

Computational time for all parameter combinations applied to simulation Setup 1 datasets was reported in the **Supplementary Figure 15**. Some of the methods scaled in time all simulated datasets dimensions while others took longer on the largest datasets (with 5000 number of cells). Note that many of the trends observed here were previously mentioned in the real datasets analysis. The fastest group of methods across all datasets dimensions include: ascend, monocle3, pcaReduce, Seurat, many combinations of sincell,sscClust when PCA dimension reduction was used and TSCAN. Other methods like CIDR, DIMMSC, Linnorm with set number of clusters and some combinations of RaceID3 and sincell were still relatively fast in time running for few minutes on datasets with the highest number of cells. SC3, Linnorm (with estimated number of clusters), sincell (when nonmetric-MDS was used as dimensionality reduction) and rest of the combinations of RaceID3 or sscClust took about one hour when applied to the largest simulated datasets whereas SIMLR computational time exceed few hours in that case being the longest method among all.

## Discussion

In this study, we evaluated the performance of several clustering methods on a wide range of real and simulated scRNAseq datasets. Such methods are distributed as open-source R packages and they constitute a significant part of the computational tools nowadays available for inferring the unknown composition of cell populations from scRNAseq data. Our comparison aimed to provide insight into the mode of usage for each of these packages depending on the structural assumptions we are willing to make. We compared the ability of the different packages to infer the unknown number of cell populations, the sensitivity of the methods across different datasets and their computational cost. For each method we tested different parameter configurations, revealing the great impact of parameter setting on the performance of individual methods. In particular, we found that some of the methods performed relatively well across most of the datasets we have considered and with respect to the different choices of the parameter settings (i.e., CIDR, and several combinations of Linnorm, SC3 and sscClust), or often poorly, as, sincell (with many parameter settings) and ascend. Other methods, such as DIMMSC, monocole3, RaceID3, Seurat, SIMLR and TSCAN, can be placed in the middle in terms of overall performance across all datasets, despite the fact that on few datasets they could have reported good performance. However, we should consider that the field of clustering of scRNAseq data is rapidly evolving. Novel methods are continuously emerging and those that we have compared are undergoing to an extensive revision that might improve their performance. It is not easy to explain why certain methods work better than others, since they perform several steps before applying the clustering algorithm. However, one of the reasons is that some methods were originally developed to analyze scRNAseq data collected under specific protocols (i.e., consisting of datasets with a limited number of cells). Then, the novel challenges (in particular the increasing size and cell heterogeneity) provided by the rapidly evolving scRNAseq technology made them not any more competitive for the complex types of data that are emerging. Reasonably, methods have to be optimized with respect to a specific protocol or dataset size, rather than attempting to find methods that work well on a wide range of scRNAseq conditions. In fact, our study showed that no methods seem to emerge as performing better than others on all datasets. Additionally, our results also showed that there is still space for improving the overall performance of the available methods on large and complex datasets or providing novel and more accurate methods.

We have found that despite different basic preprocessing options, there is no global pre-processing strategy which improves significantly the performance of all methods (packages). Instead, we found that the performance of several methods strongly depends on their parameter settings: in Seurat when varying the number of input dimensions; in SIMLR when estimating the true number of cell groups; in sincell when varying the clustering techniques and in sscClust when changing the dimension reduction step. We believe that the impact of the choice of the method-specific parameters on its performance has been underestimated till now, while it turns out to be crucial when using these methods. Unfortunately, we did not identify a golden rule for choosing the parameters. However, depending on the methods used, we identified some better performing configurations: sscClust performed better with iCor as dimensionality reduction step; Seurat with the internal choice of the number of dimensions; Linnorm and SC3 with a set number of clusters (using the true number of cell populations). On the basis of our results, we suggest that users should be more aware of the different possibilities that several methods offer in terms of parameter choices and modes of usage. Moreover, we recommend them to always evaluate the robustness of their partition with respect to changes in the parameter settings. At the same time, method developers should give more attention in better documenting all the possibilities that their methods can offer also testing their robustness with respect to changes in the settings. To this purpose, the benchmark pipeline developed for this study can be easily modified to offer an environment where other/novel methods can be evaluated.

We also observed that the poor performance of several methods/parameter combinations is often associated with a poor estimate of the number of clusters (see for instance estimation accuracy of monocle3, SIMLR or sincell). Although a rigorous assessment of the number of cell populations on real data dataset could be debated, our results show that several methods tend to significantly underestimate or overestimate the number of clusters, when compared to the true (usually unknown) cell populations. Therefore, we can say that the estimation of the number of hidden cell populations remains challenging in the scRNAseq data analysis and we hope that novel approaches will provide less biased estimates. Moreover, by comparing the performance of each method when the true number of clusters was imputed with those when it has been estimated from the data, it is possible to quantify the impact that a more accurate estimate of the number of cell populations can have on the overall accuracy.

The dataset dimension and complexity turns out to be clearly influential with respect to the running time of the methods and to the overall performance that the methods can achieve. In particular, SIMLR run time increased together with the sample size and was often the longest among other methods by several orders of magnitude (requiring up to several hours to analyze a given dataset compared to few seconds/minutes for the other methods). Similarly, scalability issues were observed in SC3, although to a less extent. In contrast, other methods/parameter combinations showed a good scalability in their computational time, as ascend, CIDR, monocle3, pcaReduce, RaceID3 (with non-internal number of dimensions), Seurat (with PCA dimension reduction), sincell, TSCAN or sscClust, limiting the computational time to few seconds/minutes. We want to stress that computational issues are becoming particularly important since modern technologies are now allowing to simultaneously sequence thousands or even tens of thousands of cells, thus it is expected that researchers will have to analyze much larger datasets. Hence, it will be important to provide novel methods that have good scalability properties either in terms of running time and/or computational resources required for their execution. This can be achieved either by designing methods with efficient algorithms and by better exploiting the parallel and high-performance computing in their implementation. From a technical point of view, we also observed frequent failures of some methods under particular cases. For example, SIMLR method failed on most of the simulated datasets with 5000 number of cells. We suspect that the method required large amounts of memory on the high-sample datasets than that available in our system. Other failures, like in monocle3, Linnorm and sincell on FPKM/RPKM datasets were related to the choices on the number of reduced dimensions. In fact, all of them encountered technical errors when used with tSNE dimension reduction and more than 3 number of dimensions. Additionally, Linnorm failed on raw and simulated datasets with a high percentage of dropouts (above 70% of zeros in the dataset) suggesting the low capacity of the method to handle high rates of missing data. Such points are probably less relevant and could be solved with future releases of the methods.

Finally, it is also worth to mention that some of the methods, such as ascend, monocle3, SIMLR, sscClust and some combinations of Linnorm and sincell, showed variability in the clusterization despite the global setting of the seed. The fluctuations can be spotted by looking at the accuracy of methods on the identical datasets across three simulation setups (see results across **Supplementary Figure 12** and **Figures 10** and **11**) or by looking at the accuracy of the methods on datasets not affected by filtering (see **Supplementary Figures 3** and **4**). We notify that the results in such cases might not be easily reproducible. In the spirit of reproducible computational research, the user should be aware of such limits.

## Conclusions

Concurrently with technical improvements in single-cell RNA sequencing, there is a rapid growth in the development of new methods, in particular, those related to the identification of cellular populations. Newly developed methods differ considerably in their computational design, implemented algorithms and available steps giving the user a large number of options to select parameters and perform a cluster analysis on scRNAseq data. However, such possibilities are often hidden and not fully documented in the software code and their impact has to be better understood.

We are not aware of any comprehensive studies aiming to test various modes of usage of the available methods on large scale datasets that have different experimental complexity in terms of dimensionality, number of hidden cell populations or levels of noise. Our benchmark approach extends the previous comparative studies (Freytag et al., 2018; Duò et al., 2018; Tian et al., 2019) to a broader range evaluation of the algorithms which depends on the parametrization (user-specified parameter choices) and previously mentioned dataset differences. The results presented here showed that the performance of the methods strongly depends on different user-specified parameter settings and that the dataset dimensionality and composition often determines the overall accuracy of the methods. Overall, this means that most of the methods lack of robustness with respect to the tuning parameters or differences among the datasets. We found that both aspects were partially ignored in the previous studies, preventing the user to better understand the potentials and limitations of each method. Although, we did not find a “golden” rule for choosing optimal parameter configurations, our study identified some model-dependent choices which were found more robust than others. Despite that, our study also showed that the overall performance is still far from being optimal. Hence, there is a need for developing novel and more accurate methods, in particular for those datasets containing a very large and heterogeneous amount of cells. Evaluating and improving clustering approaches for scRNAseq data might be beneficial for several areas of biomedical science such as immunology, cell development and cancer see for example Haque et al. (2017).

The analysis of real and simulated datasets confirmed that the high sample size and the high number of cell populations have a great impact on scRNAseq clustering methods. In particular, we found that the estimation of the number of clusters remains challenging. We confirmed these issues in several analyzed cases where the methods either under or overestimated the true number of cell populations and the simulated cell groups. In real scRNAseq applications, overestimation of the number of clusters might be just due to methods identifying previously unknown biologically relevant sub-groups. However, underestimation of the clusters means that methods failed to distinguish accurately differences between populations of cells. Since in scRNAseq clustering we also aim to identify novel and/or rare cell populations, we typically do not know the number of cell populations. The failure to identify the number of sub-groups in a consistent manner is a considerable drawback when it comes to practical applications of such methods. In fact, such failure is usually paid with a lower ARI index. By comparing the performance of each method when the true number of clusters was imputed with those when it has been estimated from the data, one can quantify the impact that a more accurate estimate on the number of groups can have on the overall performance.

With the development of new high-throughput scRNAseq protocols, the data dimensionality grows and one has to consider not only methodological performance but also computational requirements of the different approaches. We have demonstrated that computational cost does not always trade for empirical accuracy and some configurations are just unpractical for specific protocols. Since, larger and more complex datasets are going to be produced by novel droplet-based protocols, the computational feasibility needs to be better faced and more attention should be given in designing methods with efficient algorithms and in better exploiting high-performance computing in their implementation.

Taken all together, our systematic evaluation of the methods confirmed some common sense assumptions or expected results, but also identified new potential issues in scRNAseq clustering. The summary of the methods presented here can guide the readers in a number of options that the methods provide also giving awareness about their possible limitations. Moreover, the benchmark pipeline developed for this study is freely available and can be easily modified to add novel methods.

## Data Availability Statement

Publicly available datasets were analyzed in this study. This data can be found here: GSE84133, GSE65525,GSE60361, GSE67835, GSE45719, MTAB-3321, MTAB-2600, GSE81861, GSE74672, GSE71585, GSE45719, MTAB-5061, GSE71585, GSE81608, GSE36552, GSE57249, GSE52583.

## Author Contributions

MK designed and implemented the clustering benchmark study, performed both real and simulated analysis, selected and discussed results and wrote the manuscript, YR and LC contributed to the design of the benchmark study, the selection, and discussion of results and the drafting of the manuscript, AB contributed to the selection and discussion of the real and simulated data analysis and provided constructive comments on the benchmark study, CA contributed to the design of the benchmark, guided and supervised all phases of benchmark implementation, selection, and discussion of results and wrote the manuscript. All authors read and approved the manuscript.

## Funding

We acknowledge INCIPIT PhD program co-funded by the COFUND scheme (Marie Skłodowska–Curie Actions) grant agreement n. 665403, EPIGEN project and ADViSE project for financial support.

## Conflict of Interest

The authors declare that the research was conducted in the absence of any commercial or financial relationships that could be construed as a potential conflict of interest.
