# Supplementary material for: Benchmark and Parameter Sensitivity Analysis of Single-Cell RNA Sequencing Clustering Methods
Source: Front Genet. 2019 Dec 11;10:1253. doi: 10.3389/fgene.2019.01253 (PMC6918801; doi:10.3389/fgene.2019.01253)
Supplement: Supplementary file 1 [file DataSheet_1.pdf]

Supplementary Material

Brief description of the methods

For the sake of completeness, we provide a brief description of the main features of the methods we have considered in this study.

ascend (Analysis of Single Cell Expression, Normalisation, and Differential expression) is a general-purpose method for the analysis of single-cell RNA-seq data. It provides several functionalities, including those for gene filtering and normalization (here reported as `method specific` additional preprocessing). `ascend` filters genes that are expressed in a low percentage of cells and normalize expression matrix with the Relative Log Expression (RLE) approach. Before clustering, it uses PCA as an `internal` procedure for dimension reduction. The number of dimensions can be either provided by the user or a default value equal to 20 is used (`TRUE/internal`). To cluster cells, it implements an Optimal Resolution CORE algorithm which is an iterative hierarchical clustering applied to the Euclidean distance matrix calculated between the cells. Finally, it estimates the number of clusters through the split and merge algorithm over multiple iterations of the clustering process.

CIDR (Clustering through Imputation and Dimensionality Reduction) is a clustering method designed to deal with dropouts present in single-cell RNA-seq data through imputation. Firstly, the method identifies dropout candidate genes and estimates the relationship between dropout rate and gene expression levels to impute zero values. Note that it does not provide any specific data filtering or normalization functions (`none`). Then, it applies Principal coordinate analysis (PCoA) on the imputed gene expression levels, as an `internal` procedure of dimension reduction. PCoA is very similar to the PCA dimension reduction technique except that instead of using a correlation matrix it uses a cell distance matrix. Hence, the low dimensional representation approximately preserves cell proximities in the analyzed dataset. The number of dimensions can be either provided by the user or a default value equal to 4 is used (`TRUE/internal`). Afterward, it clusters the data using a fixed hierarchical clustering (based on Ward's algorithm). It allows to set or estimate the number of clusters (`set/estimate`). For the estimation, the method uses Calinski-Harabasz index (ratio of between and within-cluster variances).

DIMMSC uses Dirichlet Mixture Model to cluster single-cell RNA-seq datasets produced by droplet-based protocols. In contrast to other methods, it does not provide any specific functionalities for additional preprocessing or dimension reduction (both options `none`), but it models directly the gene expression matrix. To model data, the method uses a `fixed` clustering technique that assumes each cell expression follows a multinomial distribution. Variations across different cell clusters are characterized via Dirichlet mixture priors. Note that `DIMMSC` does not estimate the number of clusters thus only option `set` is available.

Linnorm is mainly a normalization and transformation method designed to improve the analysis of scRNAseq data. The normalization is performed using batch effect and library size correction. The user can choose if to use such additional `method specific` preprocessing step or not (`none`). Then, the method applies PCA or tSNE dimension reduction followed by a fixed clustering technique (K-Means) or, alternatively, hierarchical clustering in the original data space. When using PCA or tSNE one can set the number of reduced dimensions or use an internal value (3 dimensions) (`TRUE/internal`). Similarly, with a fixed clustering technique one can either set or estimate the number of clusters (options `set/estimate` available). Cluster number estimation is performed using four packages `fpc`, `vegan`, `mclust` and `apcluster`. Note that when hierarchical clustering is applied this option is not available (`none`). Before, applying any type of above-mentioned clustering, the method first transforms data

towards homoscedasticity and normality using a modified version of the logarithm transformation. In theory, such a transformation should provide stronger noise elimination. Note that this step of the algorithm is not optional.

monocle3 is a general-purpose tool for the analysis of single-cell gene expression data that provides to the user several functionalities not only to preprocess and cluster scRNAseq datasets, but also a wide series of other downstream procedures. For example, it allows to use or not an additional preprocessing functions (`none/method specific`). The method-specific additional preprocessing consists of log-transformation of the counts. Then it provides two different algorithms for dimension reduction, (`tSNE` and `UMAP`). In both cases, the number of dimensions can be either provided by the user or a default value equal to 2 is used (`TRUE/internal`. In particular, UMAP (Uniform Manifold Approximation and Projection) uses a manifold learning technique to build two topological representations of data in low and high dimensional space. Then it optimizes the low dimensional projection by minimizing the cross-entropy between two topological representations. After dimension reduction, it provides two clustering algorithms: `densityPeak` and `louvain`. DensityPeak partitions cells based on the cell local density and their nearest distance. It aims to group cells with a higher local density and distance smaller than a fixed threshold. The number of clusters in this technique is automatically estimated through the inference. On the contrary, Louvain approach is based on a community detection algorithm that seeks densely connected modules in a graph built on the scRNA-seq dataset. Although it is possible to fix the number of nearest neighbors when creating the graph, and this value has an effect on the resolution of clustering, the number of clusters cannot be directly controlled. Therefore we classified it as `estimate`.

pcaReduce is an agglomerative method that combines PCA dimension reduction (note as `internal` technique) and K-Means clustering technique (note as `fixed`) through an iterative process. It does not implement any additional preprocessing (`none`). It starts by reducing the dimensionality of the dataset by projecting it into q-dimensional space through PCA, then uses K-Means clustering to partition cells into $q+1$ clusters. After that, for each pair of clusters, the probability of those clusters to be merged is computed. Clusters with the highest probability are joined together resulting in q clusters and dimension that explains the lowest variance is removed. This operation is repeated until only two cluster remains. The method requires to specify the initial value of q that is usually chosen relatively high to allow for finding small cell populations. As a final result, the algorithm outputs partition for each cluster cardinality from 2 to $q+1$. The user can `set` the desired number of clusters.

RaceID3 (Rare Cell Type IDentification) method has been developed to enable the identification of rare and abundant cell types from single-cell RNA-seq data. Before applying the main procedures, the method requires to additionally preprocess the data through filtering and normalization (denoted as `method specific` preprocessing). The filtering of genes is done according to a minimum expression threshold (minimum expression = 5) in a given number of cells (number of cells = 1). Then, the count matrix is normalized (to the minimum of a total expression across all the cells) and its dimension is reduced with PCA dimension reduction either with a fixed number of dimensions or internal that is estimated via saturation criterion procedure (both `TRUE/internal` options available). Formally, within the method there is also ICA dimension reduction, however, we discarded it due to technical errors obtained in all clustering attempts. After dimension reduction is performed, cells can be clustered with one of the three clustering techniques: K-means and K-medoids or Hierarchical clustering (denoted as `k-means`, `k-medoids`, `hclust`). Each type of clustering can be used with a set or estimated number of clusters (`set/estimate`). Estimation is done through the saturation criterion procedure that tries to find a saturation point in the mean within-cluster dispersion as a function of the cluster number.

SC3 is a consensus clustering method for single-cell RNA-seq data. It offers an additional preprocessing step (herein `method specific`) in terms of gene filtering and log-transformation. Alternatively it can work without it (option `none` available). When using preprocessing step, it firstly removes the genes with a percentage of dropouts smaller or higher than a given threshold (minimum number of dropouts = 10, the maximum number of dropouts = 90), then it log-transforms the counts. Afterward, the method calculates distances between the cells using three metrics: Euclidean, Pearson, and Spearman. On each of the obtained distance matrix two transformations are applied: PCA and Laplacian graph (we called this procedure as `internal` in the place of dimensionality reduction). Note that the user cannot select the number of reduced dimensions (only `internal` option available). Afterward, a fixed clustering technique (K-Means) is used to cluster the transformed distance matrices subject to the first `d` eigenvectors. In brief, several individual clusterings are obtained which are further combined into a single consensus clustering using the Cluster-based Similarity Partitioning Algorithm (CSPA). In this clustering approach, only a set number of clusters is a valid option (`set`).

Seurat method contains various procedures for quality control, analysis, and exploration of single-cell transcriptomic data. At first, the method requires to use an additional `method specific` preprocessing by filtering genes detected in a specified number of cells (the internal setting for the number of cells = 5) and normalizing the full count matrix by its total count that is multiplied by a scaling factor. Then `Seurat` log-transforms the data and offers two dimension reduction techniques to be applied: PCA or ICA (Independent Components Analysis), both with a user-specified or the internal number of reduced dimensions (`TRUE/internal`) (the internal value is equal to the full number of features). Then, on the data reduced space, the K-nearest neighbor (KNN) graph is constructed (based on the Euclidean distances between any pair of cells) and weighted by the shared overlap in the local neighborhoods of the nodes. `Seurat` to cluster cells represented in such graph structure, uses modularity detection Louvain algorithm (herein called `fixed`) that estimates the number of clusters by optimizing the modularity function (Butler et al., 2018).

SIMLR core algorithm lays in the novel cell-to-cell gene expression similarity measure (Single-cell Interpretation via Multi-kernel LeaRning) that aims to effectively cluster the cells obtained from any scRNAseq platform or biological experiment. Prior applying the algorithm `SIMLR` allows to use or not (options `method specific` or /textttnone) an additional preprocessing which substitutes the counts with the mean expression of each gene. Then, the method adopts an optimization framework with multiple kernels (each of which represents a distance measure) to construct the distances between the cells. `SIMLR` assumes that if there are C populations within cells the similarity matrix should have an approximate block-diagonal structure with C blocks that show the largest similarities. Afterwards, on the cell-to-cell similarity matrix, `SIMLR` applies tSNE dimension reduction either with a set or internal number of dimensions (options `TRUE/internal`). Then, a fixed clustering technique (in this case K-Means) is used to partition cells into a number of cell populations either specified by the user or estimated through the internal procedure (`set/estimate`). When using a specified number of clusters, the same value will be used also as the internal number of dimensions. Otherwise, the algorithm will find itself the most optimal value for the number of dimensions.

sincell method extends standard approaches for scRNAseq data analysis by providing several options to reduce the dimension of the data and to cluster the cells. `sincell` does not offer any explicit functions for data additional preprocessing however when initializing the method, it filters out the genes with zero variance across all the cells. After that, the method gives five different options for reducing dataset dimension including PCA, ICA, tSNE, classical-MDS, and nonmetric-MDS that can be either used

with a provided number of dimensions or an internal setting (2 dimensions) (options TRUE/internal available). Classical-MDS, also known as Principal Coordinates Analysis (PCoA) tries to find the low-dimensional embedding based on pairwise distances between the cells. Nonmetric-MDS searches for the low-dimensional representation of cells that highly preserves distances in the original space however by use of a less demanding relationship between dissimilarities and cell-to-cell distances (for more details see (Cox and Cox, 2000)). Afterward, the clusterization in sincell can be done with one of the five techniques: K-Medoids, Hierarchical clustering (ward.D option), Maximum distance (max.distance), percent and knn. K-Medoids is a similar technique to K-Means however it selects for a cluster centers the points that belong to the dataset. Hierarchical clustering, on the other side, builds the hierarchy of the clusters based on pairwise distances between the cells and cuts the tree at a particular number of cell groups (internally set up to 3). When Maximum distance or Percent are used, the algorithm first creates a totally connected graph from which the connections lower than a given threshold are removed or a percent of shortest pairwise distances are kept, respectively. On the contrary, the KNN technique starts from a totally disconnected graph subsequently connecting each cell to a number of nearest neighbors (internally set to 3). Note that in sincell method when using any of the clustering techniques there is no possibility to explicitly set the number of cell groups, thus only estimate option is available.

sscClust method provides several options for clustering single-cell RNA-seq data. Although it does not give any explicit procedures for data additional preprocessing (thus we call the preprocessing option none), we are aware that the method filters and normalize the matrix internally (although it is not an optional step for the user). sscClust allows to reduce data dimension with one of two techniques: PCA or Correlation (iCor). If iCor is used then the method calculates the correlation between samples (default correlation is Spearman) that is followed by tSNE dimension reduction. Both dimension reduction techniques can be used with internal procedures (based on "elbow" method) for determining the number of reduced dimensions (option internal) and only PCA allows to set this value by the user (option set). Afterwards, one can choose between four clustering techniques: k-means, ADPclust, hclust and SNN. All of them except SNN can be used with set of estimated number of clusters. SNN technique creates a Shared Nearest Neighbors graph that links only nodes (here cells) which are in the nearest neighbor of each other (determined by some threshold). Then modularity detection algorithm is applied to find densely connected nodes (cells clusters). Note that this technique does not allow to explicitly set the number of clusters.

TSCAN is mainly a pseudo-temporal ordering method that also incorporates procedures for clustering or testing differentially expressed genes. It first performs a required additional preprocessing step (only method specific option available) by taking the logarithm of the counts and filtering lowly expressed genes (based on the zero-proportion) that additionally have a low coefficient of variance. Then, the method performs PCA dimension reduction with the internal number of dimensions (here both options called internal)). Clustering is done via fixed model-based technique that fits a mixture model to the dataset (a mixture of multivariate normal distributions) where each component has a variance-covariance matrix that follows 'ellipsoidal, varying volume, shape and orientation' (for more information see (Fraley and Raftery, 2002)). Then, for each cell, a posterior probability is computed that tells how strongly the cell belongs to a particular cluster. Cells with the highest probabilities are then assigned to the corresponding clusters. The mentioned clustering procedure can be used either with a specified number of clusters or estimated via the Bayesian Information Criterion (BIC) (set or estimate).

8.1 Technical issues

Some methods/parameter combinations in some cases encountered technical errors during their execution (see error messages in Supplementary Table 3). Overall, we distinguish errors related to the limited resources in terms of memory that resulted in aborting the R session (i.e., as for `Seurat` and `SIMLR` on raw and simulated counts), errors that were related to the execution of a specific function (i.e., as for `DIMMSC` and `Linnorm` on raw counts) or as a result of applying a specific parameter setting (i.e., as when using more than 3 dimensions in `Linnorm` or `monocle3` on FPKM/RPKM counts). Methods that aborted R session when analyzing large datasets possibly require larger amounts of memory than those provided in our machine. Errors related to the specific parameter setting (as for combinations used with more than 3 dimensions) suggest using these methods with internal settings. Other types of errors were specific to the analyzed cases i.e., `Linnorm` failed mostly on Raw counts and Simulated counts with a high percentage of dropouts suggesting the low capacity of this method to handle high rates of missing data.

Additional references

Butler, A., Hoffman, P., Smibert,P. , et al. Integrating single-cell transcriptomic data across different conditions, technologies, and species. Nat Biotechnol 36, 411–420 (2018) doi:10.1038/nbt.4096

Cox, T.F., and Cox, M.A.A., (2000). "Multidimensional Scaling," in Chapman & Hall/CRC Monographs on Statistics Applied Probability, 2nd ed. (Taylor Francis) New York . doi: 10.1201/9781420036121

Fraley, C., and Raftery, A. (2002) Model-based clustering, discriminant analysis, and density estimation. Journal of the American Statistical Association, 97, 458 , 611-631. doi: 10.1198/016214502760047131.

Supplementary Tables

Dataset	Nr cells	Nr cells after QC	Nr genes	Nr genes after QC	Nr genes after QC & FILT
Baron2016_m*	1886	1886	14878	14861	14861
Klein2015*	2717	2712	24175	24047	24047
Zeisel2015*	3005	3002	19972	19972	19972
Darmanis2015	466	463	22088	21630	21625
Deng2014_raw	268	248	22958	21297	21267
Goolam2016	124	119	41480	28147	28096
Kolodziejczyk2015	704	674	38653	32225	32133
Li2017	561	519	55186	43055	42977
Romanov2016*	2881	2880	24341	21143	21143
Tasic2016_raw	1679	1574	24150	21617	21464

Supplementary Table 1. Size (i.e. number of cells and number of genes) of 10 real datasets (Raw UMI counts and Raw read counts) before/after quality control and after filtering. * Datasets not affected by filtering (equal number of genes before and after filtering).

Dataset	Nr cells	Nr cells after QC & FILT & HVG	Nr genes	Nr genes after QC & FILT & HVG
Deng2014_rpkm	268	217	22958	955
Segerstolpe2016	3514	2931	25525	1226
Tasic2016_rpkm	1679	1579	24057	542
Xin2016	1600	1583	39851	1935
Yan2013	90	90	20214	1963
Biase2014	56	52	25737	829
Treutlein2014	80	79	23271	1380

Supplementary Table 2. Number of cells and genes of 7 real datasets (FPKM/RPKM counts) before/after quality control, filtering and highly variable gene selection.

Data type	Method	Parameter setting	Error message
Raw counts	DIMMSC	several	EM_multinomial: sort_index(): detected NaN
Raw counts	Linnorm	several	Given the current minNonZeroPortion threshold, the number of remaining feature (less than 200) is too small.
Raw counts	SIMLR	several	R session aborted
Raw counts	sincell	ICA dimension reduction	fastICA: svd on 2122822416 x 433555684 exceeds Fortran indexing limits
Raw counts	RaceID3	several	switch(Z$ifault, stop("empty cluster: try a better set of initial centers"
Raw counts	Seurat	several	R session aborted
FPKM/RPKM counts	Linnorm	PCA dimension reduction	Given the current minNonZeroPortion threshold, the number of remaining feature (less than 200) is too small.
FPKM/RPKM counts	Linnorm	tSNE dimension reduction, Nr dimensions: 3	Linnorm.Norm: in if (Slope <0) { : missing value where TRUE/FALSE needed
FPKM/RPKM counts	Linnorm	tSNE dimension reduction, Nr dimensions: 5, 10, 15	Error in .check_tsne_params(nrow(X), dims = dims, perplexity = perplexity, : dims should be either 1, 2 or 3
FPKM/RPKM counts	monocle3	tSNE dimension reduction, Nr dimensions: 5, 10, 15	Error in .check_tsne_params(nrow(X), dims = dims, perplexity = perplexity, : dims should be either 1, 2 or 3
FPKM/RPKM counts	sincell	tSNE dimension reduction, Nr dimensions: 5, 10, 15	Error in .check_tsne_params(nrow(X), dims = dims, perplexity = perplexity, : dims should be either 1, 2 or 3
Simulated counts	Linnorm	several	Given the current minNonZeroPortion threshold, the number of remaining feature (less than 200) is too small.
Simulated counts	SIMLR	several	R session aborted

Supplementary Table 3. Error messages of the methods and the failing parameter settings. Reported errors occurred either across several parameter combinations of the method or only in a particular setting. Failing cases are reported in Supplementary Figures 3, 4, 6, 10 as marked in grey. We distinguish errors that were caused by a limited memory resources and resulted in aborting the R session (i.e., as for `Seurat` and `SIMLR` on raw and simulated counts) or errors that were related to the execution of a specific function (i.e., as for `DIMMSC` and `Linnorm` on raw counts) or as a result of applying a specific parameter setting (i.e., as when using more than 3 dimensions in `Linnorm` or `monocle3` on FPKM/RPKM counts).

Supplementary Figures

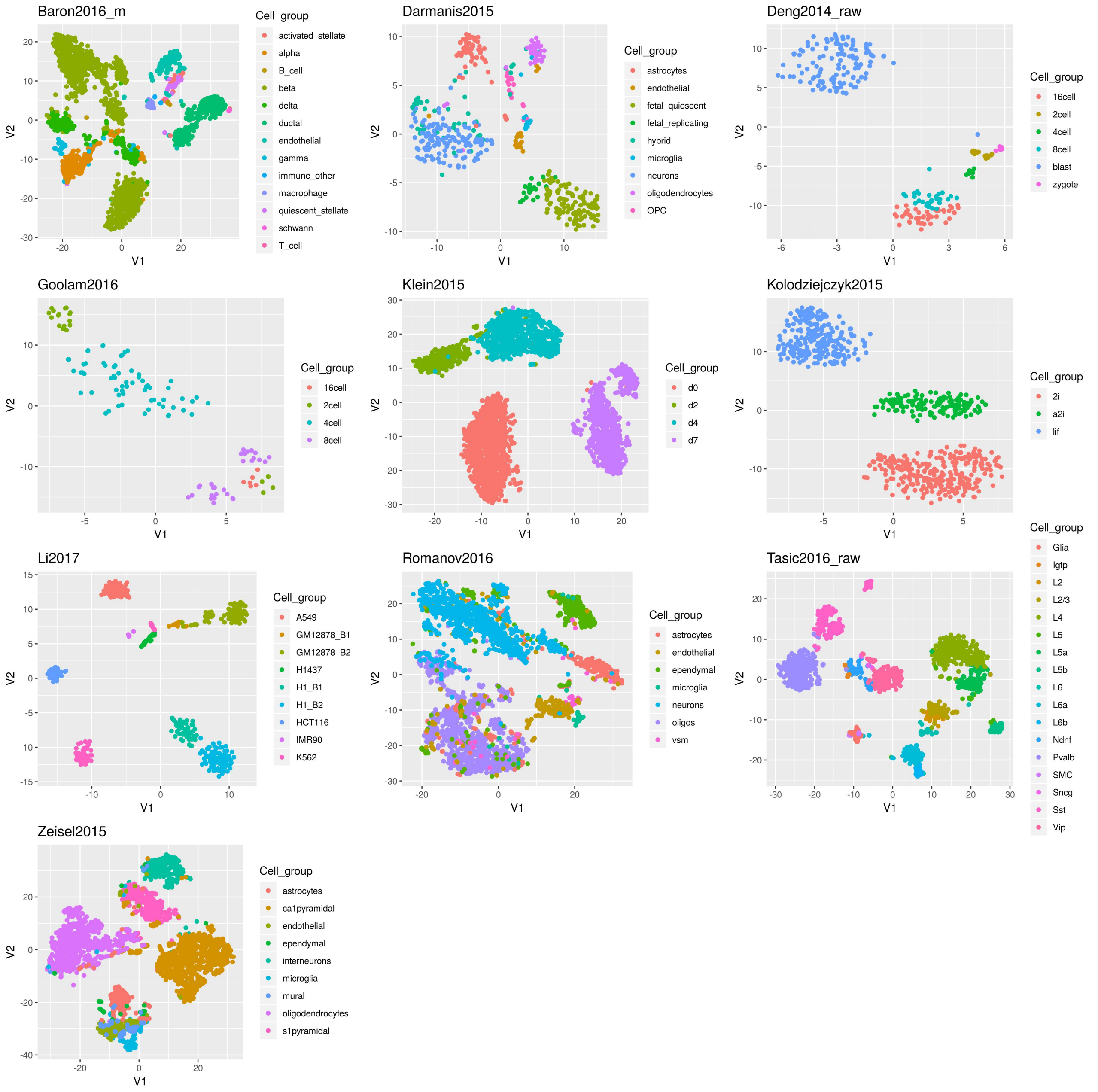

Supplementary Figure 1. One realization of the tSNE projections of the 10 raw datasets after quality control. Projections were obtained by applying tSNE dimension reduction on raw read and raw UMI counts after quality control and no further preprocessing. Dataset projections were colored by the annotated cell groups from the corresponding studies (see Table 2).

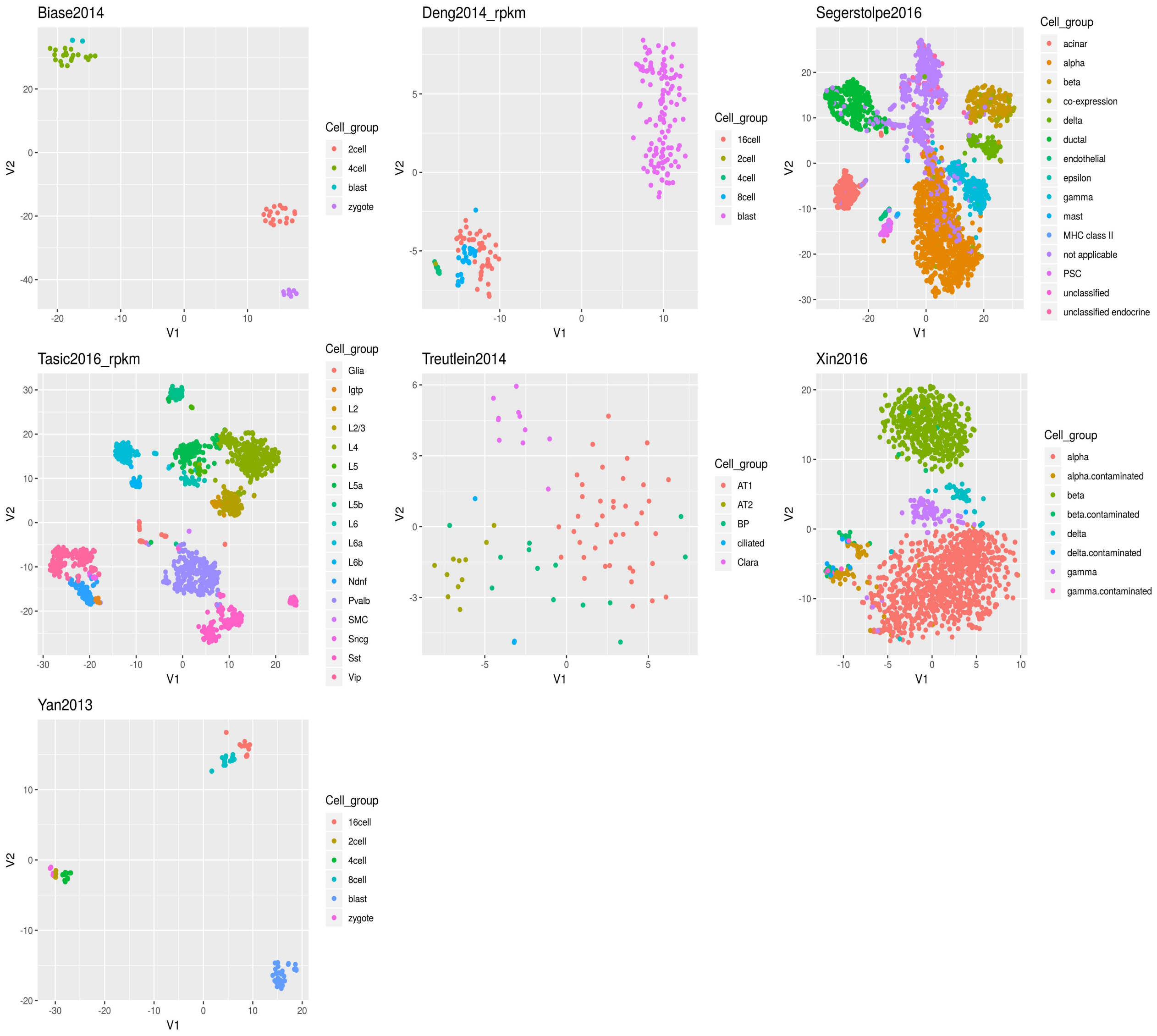

Supplementary Figure 2. One realization of the tSNE projections of the 7 FPKM/RPKM datasets after quality control and basic preprocessing. Projections were obtained by applying tSNE dimension reduction on FPKM/RPKM counts after basic preprocessing. Dataset projections were colored by the annotated cell groups from the corresponding studies (see Table 2).

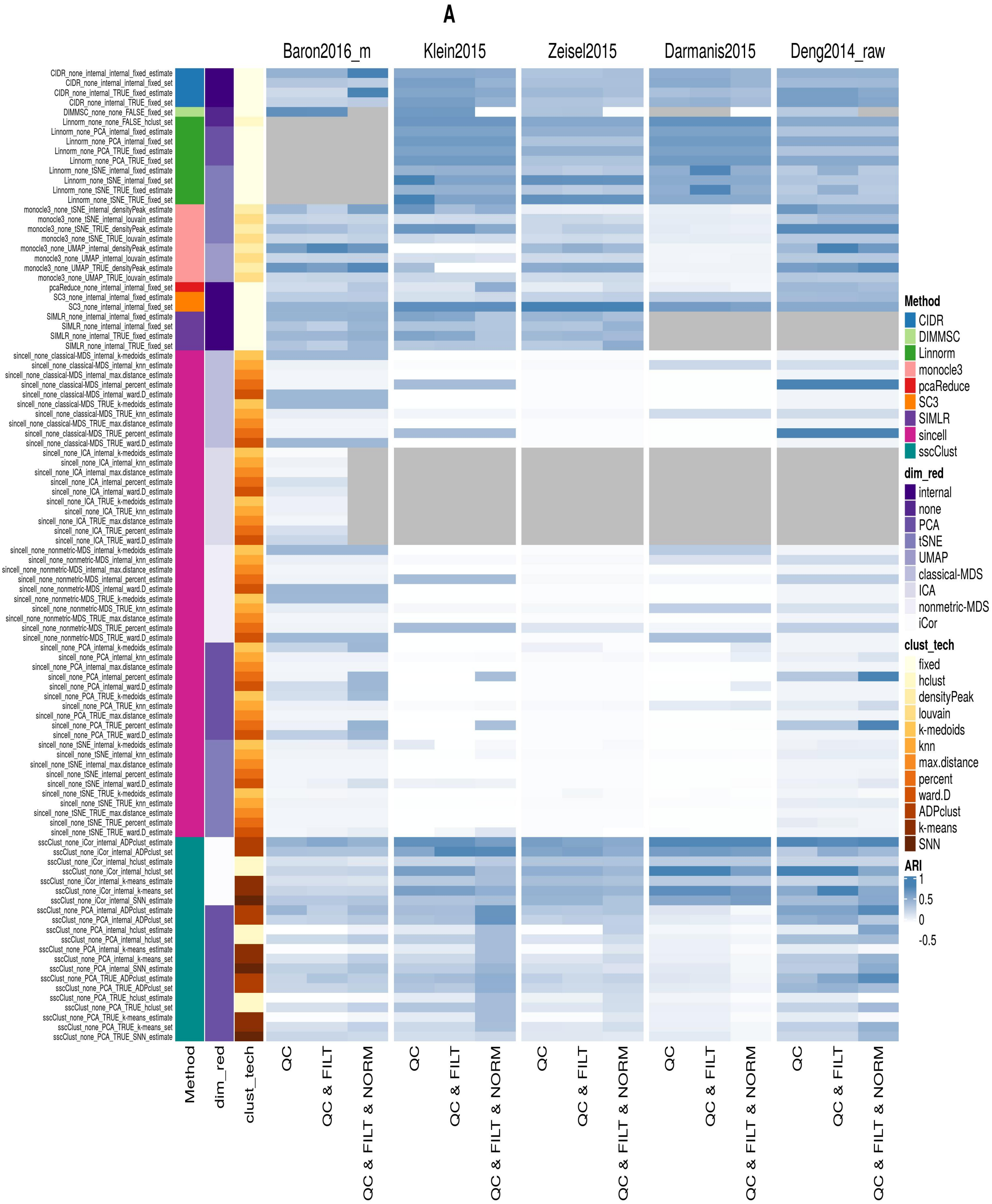
A
Baron2016_m
Klein2015
Zeisel2015
Darmanis2015
Deng2014_raw
Method
CIDR
DIMMSC
Linnorm
monocle3
pcaReduce
SC3
SIMLR
sincell
sscClust
dim_red
internal
none
PCA
tSNE
UMAP
classical-MDS
ICA
nonmetric-MDS
iCor
clust_tech
fixed
hclust
densityPeak
louvain
k-medoids
knn
max.distance
percent
ward.D
ADPclust
k-means
SNN
ARI
1
0.5
0
-0.5
Method
dim_red
clust_tech
QC
QC & FILT
QC & FILT & NORM

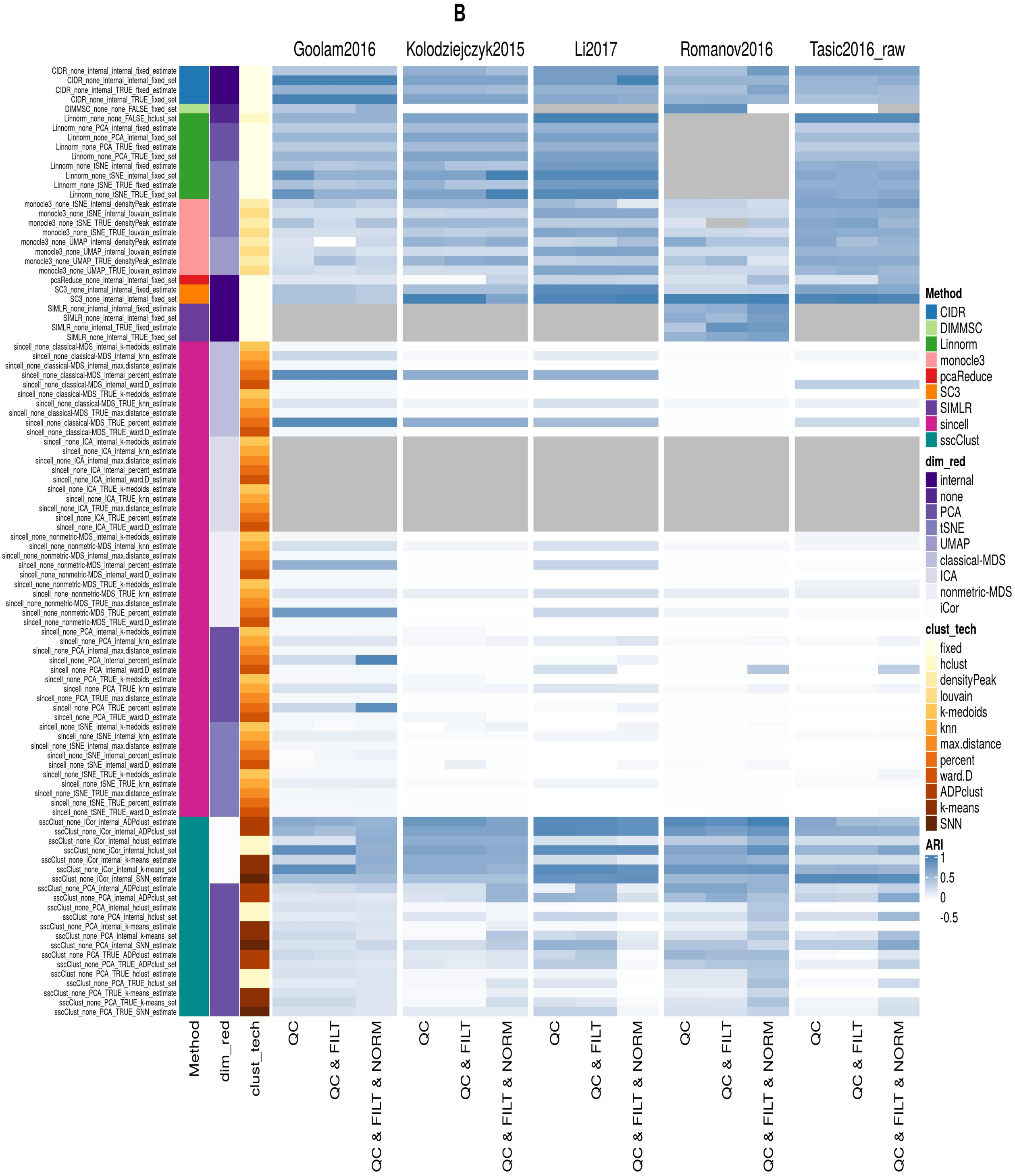

Supplementary Figure 3. Heatmap of methods performance across Raw datasets. Two heatmaps of the performance of 9 methods with 100 parameter combinations subject to `none` preprocessing, applied to 10 raw datasets, split into two panels **(A)** and **(B)**, which underwent three basic preprocessing types: QC, QC & FILT, QC & FILT & NORM. The intensity of the color (blue/white) represents the accuracy in clusterization (measured in ARI). Grey boxes stands for missing results. Left annotation indicates which dimension reduction (dim_red) and clustering technique (clust_tech) (see in the right legend) have been used in which parameter combination.

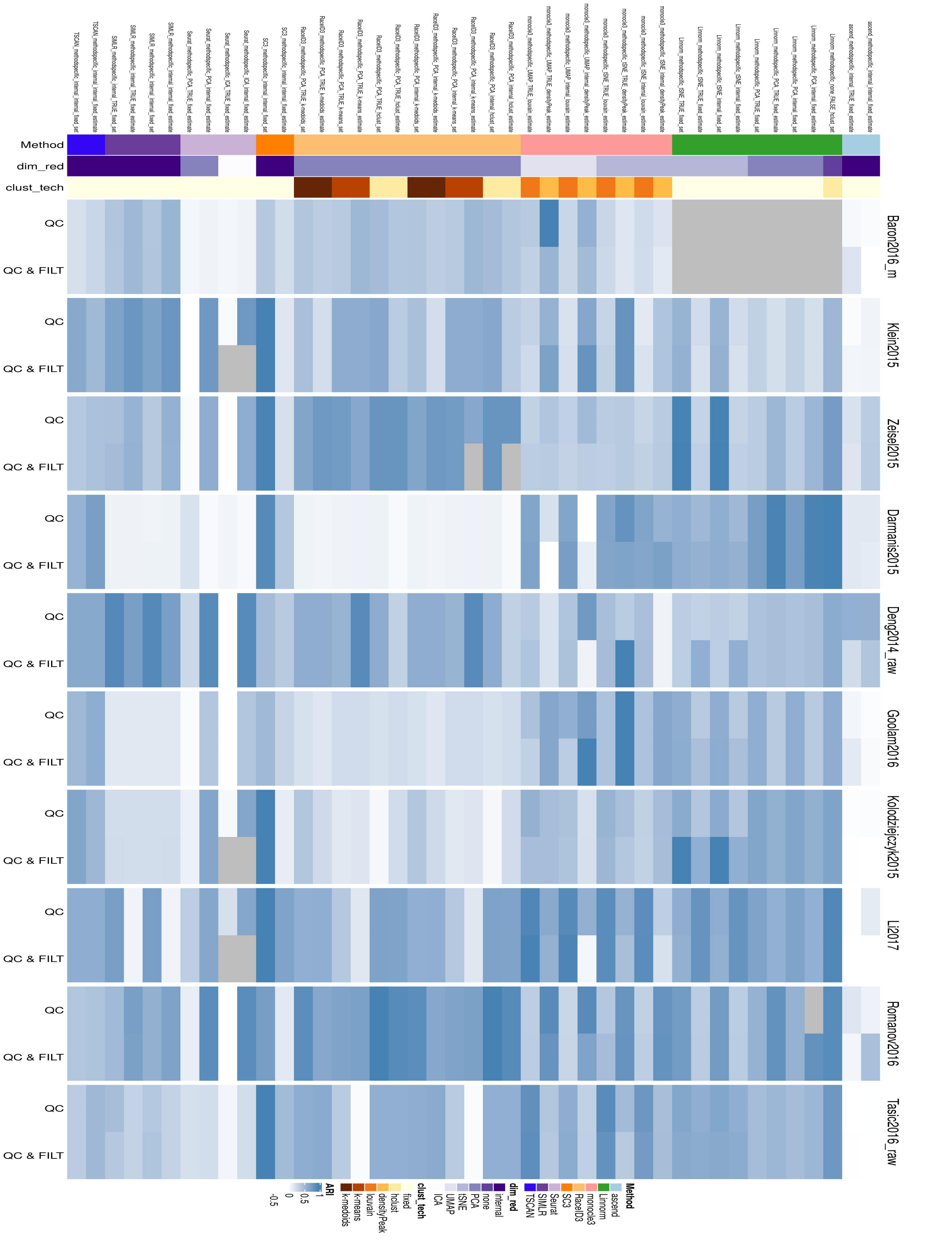

Supplementary Figure 4. **Heatmap of methods performance across Raw datasets.** Heatmap of the performance of 8 methods (43 parameter combinations with `method specific` preprocessing) applied to 10 raw datasets, which underwent two basic preprocessing types: QC, QC & FILT. The intensity of the color (blue/white) represents the accuracy in clusterization (measured in ARI). Grey boxes stands for missing results. Left annotation indicates which dimension reduction (dim_red) and clustering technique (clust_tech) (see in the right legend) have been used in which parameter combination.

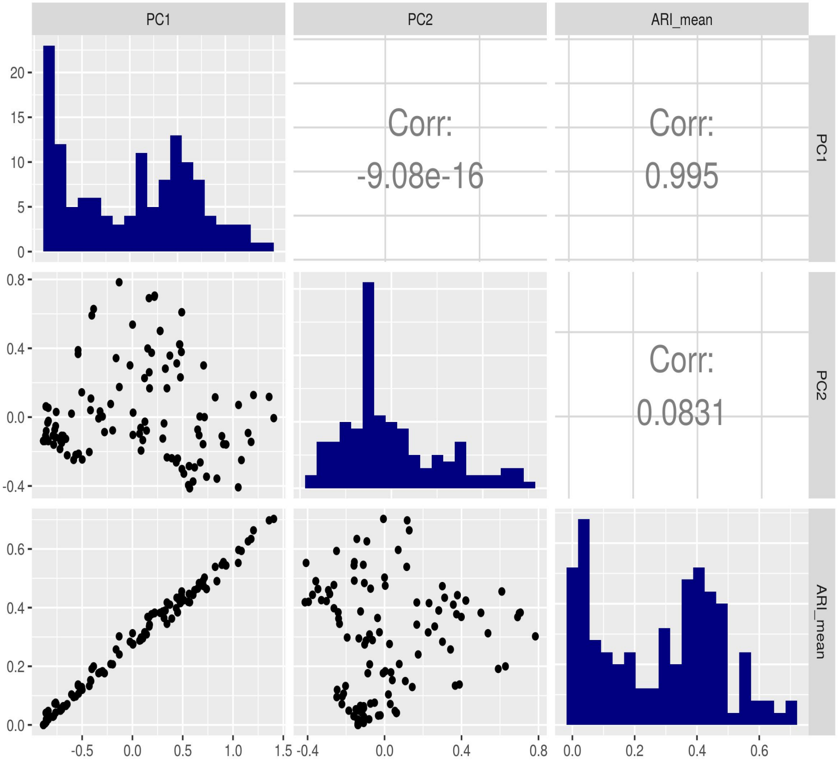

Supplementary Figure 5. Correlation between two first principal components and the ARI averaged across all QC & FILT raw datasets. PC1 is positively correlated with averaged ARI (Pearson correlation = 0.995) wheres PC2 shows very weak positive correlation with the accuracy (Pearson correlation < 0.1).

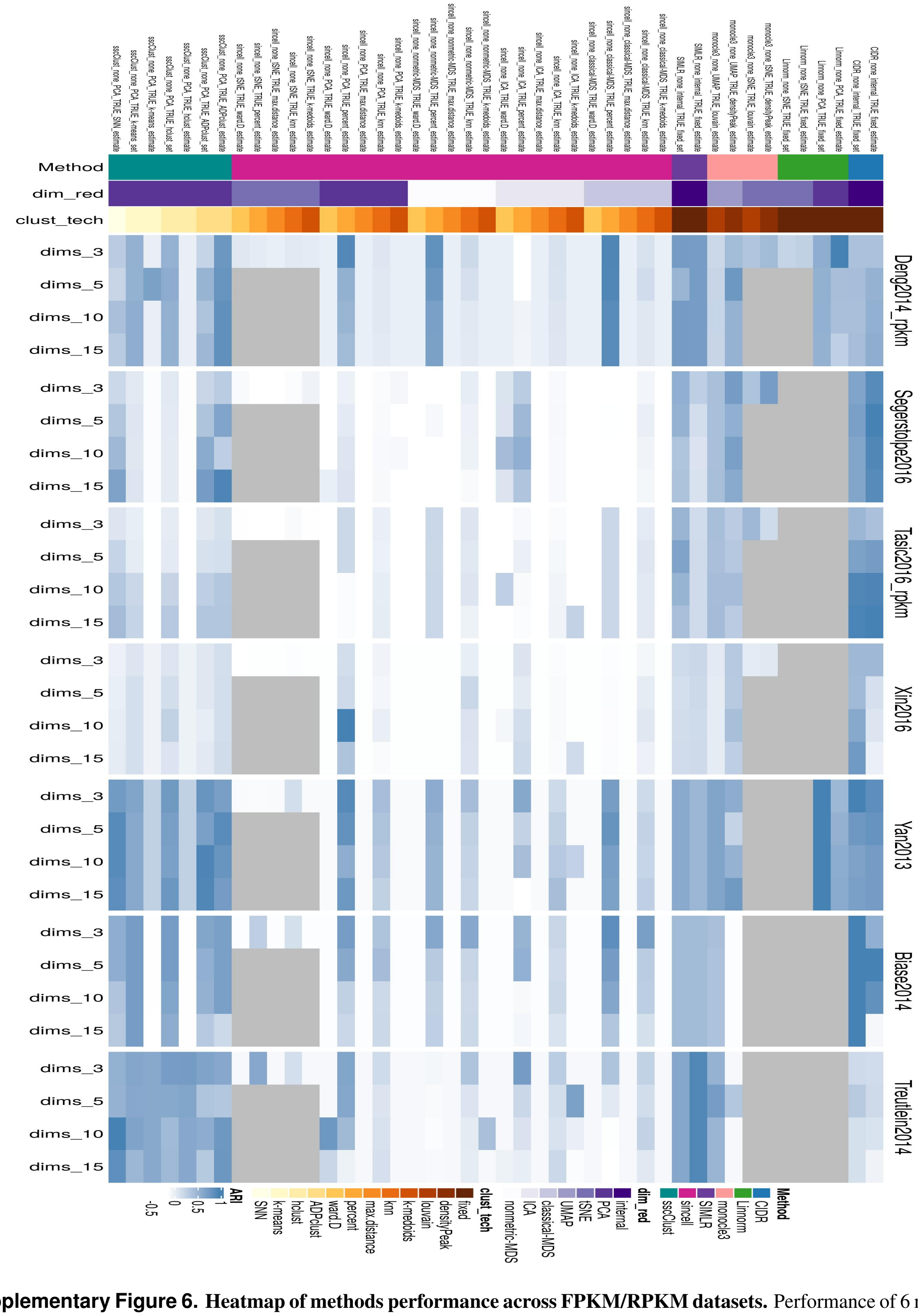

Supplementary Figure 6. Heatmap of methods performance across FPKM/RPKM datasets. Performance of 6 methods with 44 parameter combinations on 7 FPKM/RPKM datasets. The selected methods do not provide additional `method specific` preprocessing and allow for setting number of reduced dimensions.

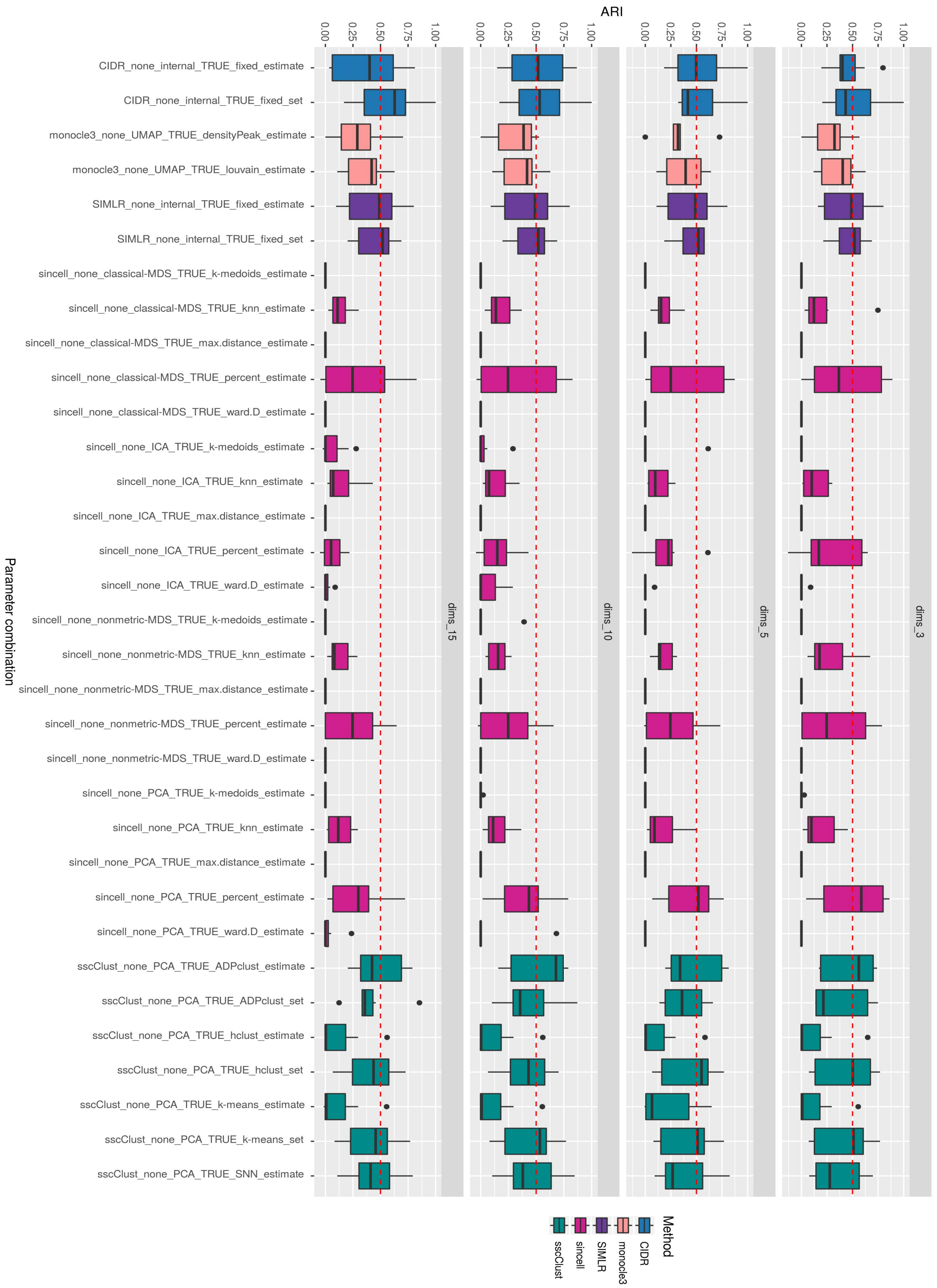

Supplementary Figure 7. Overall accuracy of methods applied to FPKM/RPKM counts. Performance of 5 methods (33 parameter combinations out of 44) on 7 FPKM/RPKM datasets. The selected methods are those that do not provide additional `method specific` preprocessing and allow for setting number of reduced dimensions.

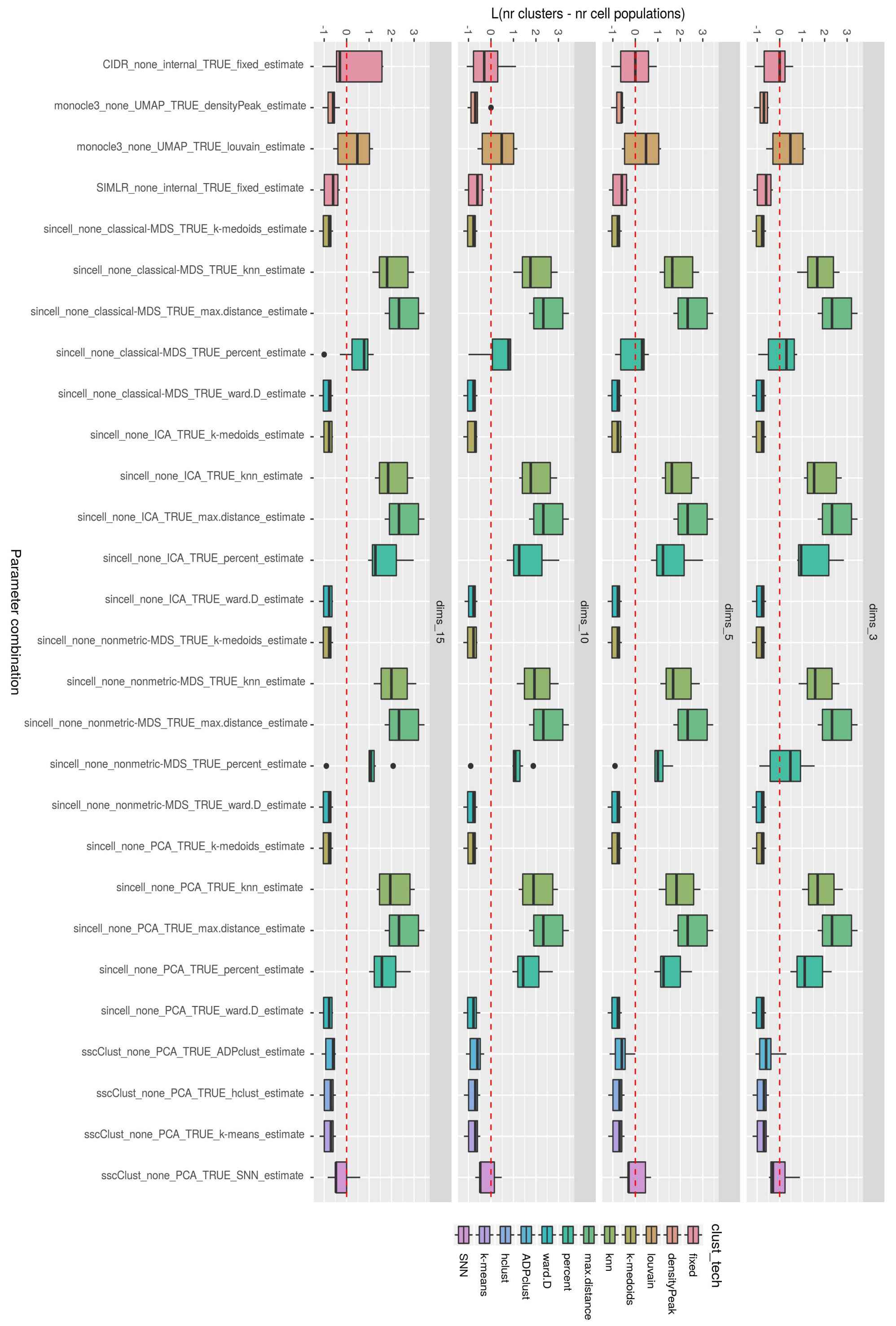

Supplementary Figure 8. **Estimation of the number of clusters for methods applied to FPKM/RPKM counts.** Boxplots of L in Eq. 1 for the subset of methods (28 parameter combinations out of 37) with `none` preprocessing, that allows to set number of dimensions (`TRUE`) and `estimate` number of clusters. We superimposed as reference, a red dashed line at L=0. Parameter combinations with difference below or above 0 resulted in under or overestimation of the number of clusters.

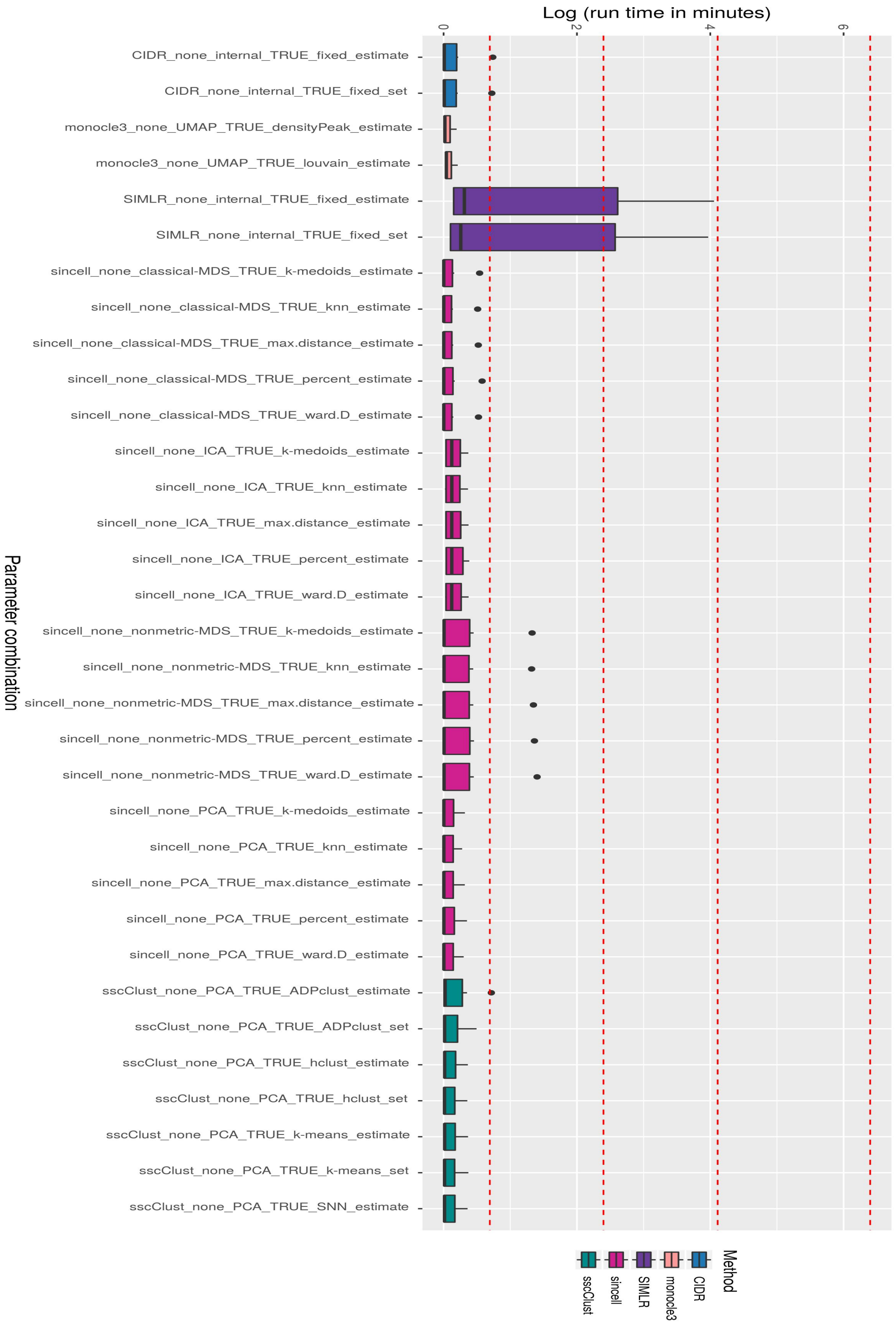

Supplementary Figure 9. Computational time of methods applied to FPKM/RPKM datasets. Log of run times in minutes of 5 methods with 33 parameter combinations applied to 7 FPKM/RPKM datasets. The selected results are those with the number of reduced dimensions set to 3. We superimposed as reference, red dashed lines at log of 1 min, 10 min, 1 hour and 10 hours.

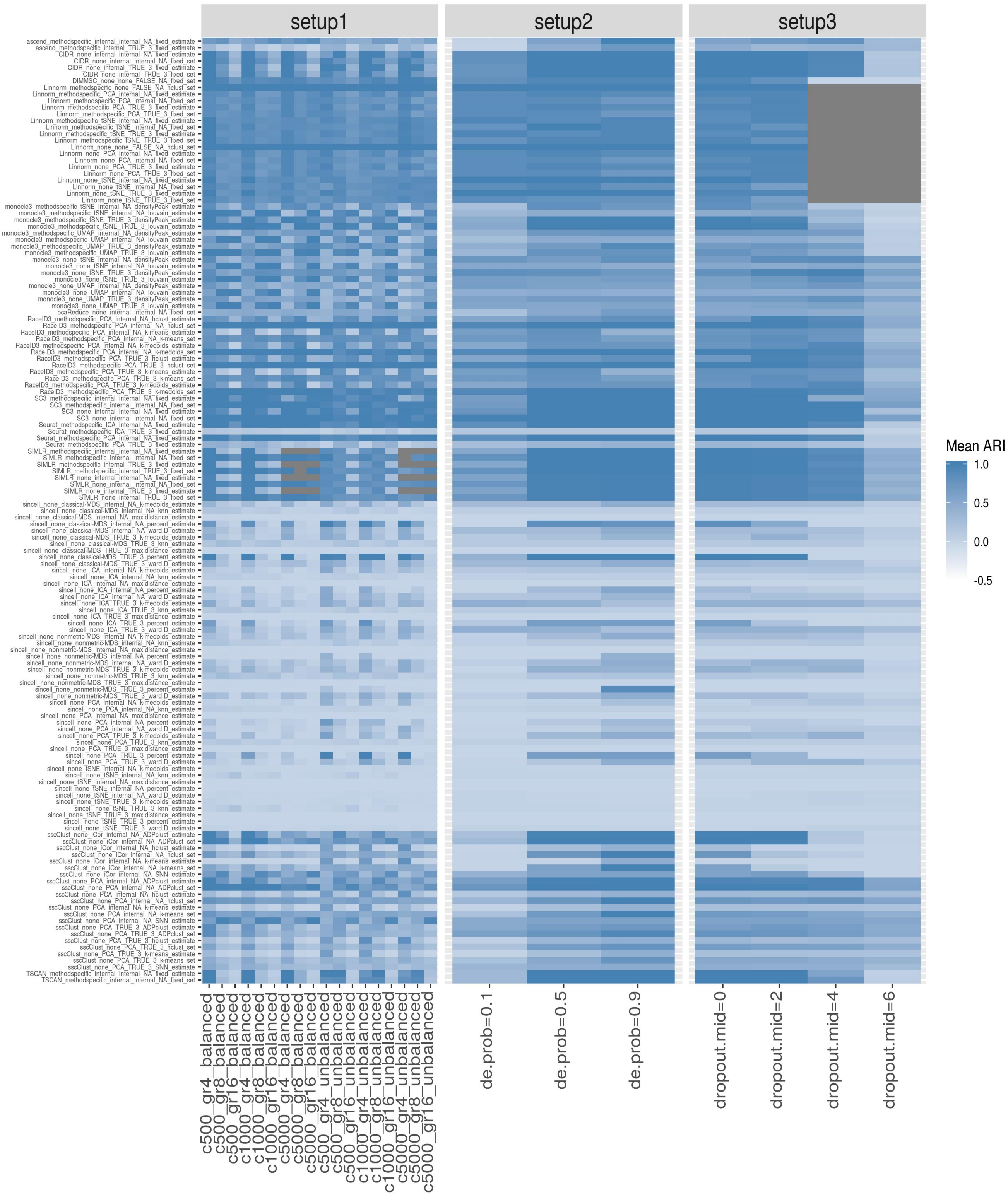

Supplementary Figure 10. Heatmap of methods performance across simulated datasets. Performance in ARI of 143 parameter combinations on simulated datasets. Reported results were averaged across all runs.

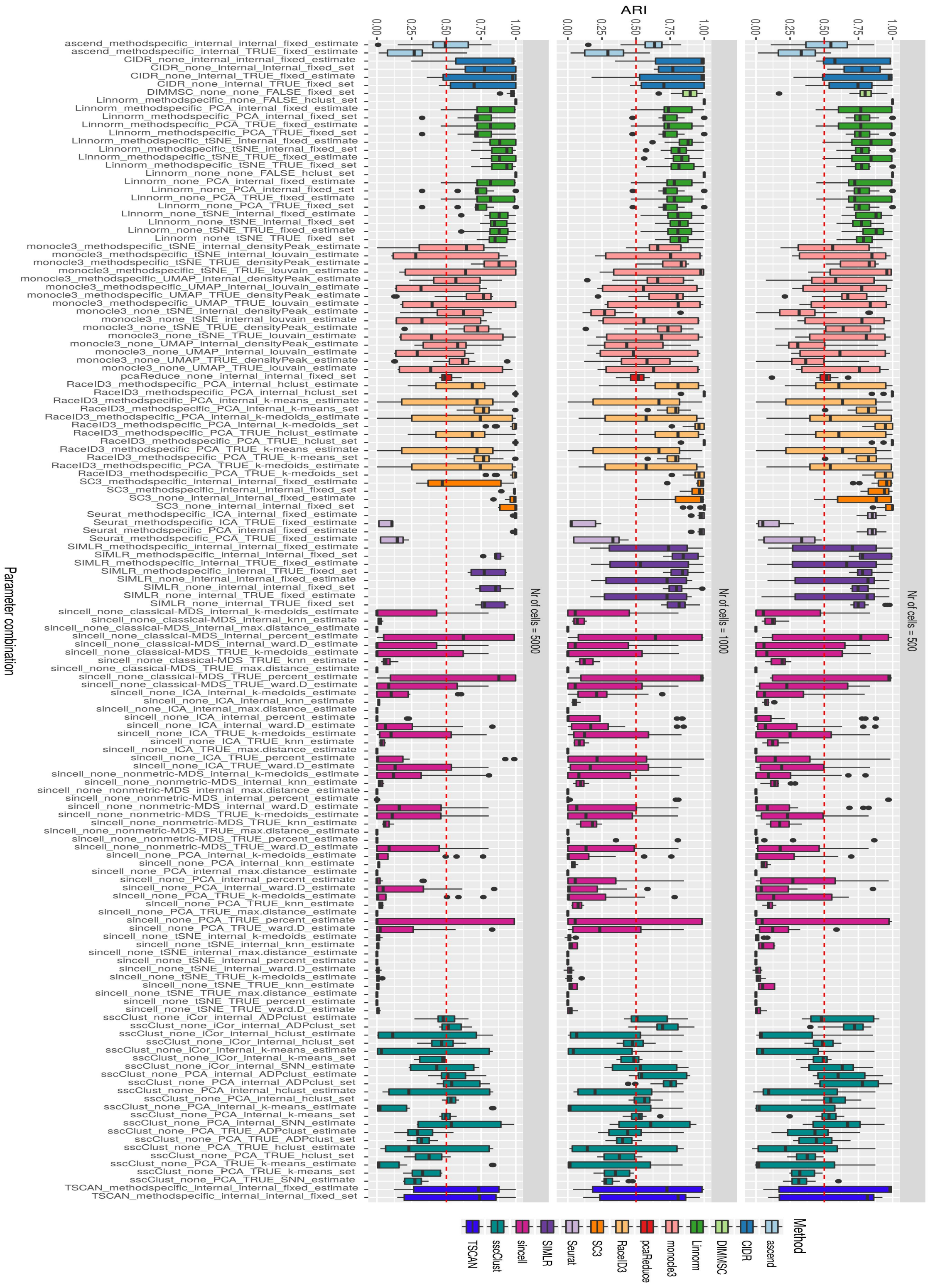

Supplementary Figure 11. Overall accuracy of the methods on simulated datasets from Setup1 with unbalanced group sizes. Performance of 143 parameter combinations on simulated datasets. Reported results are across all runs.

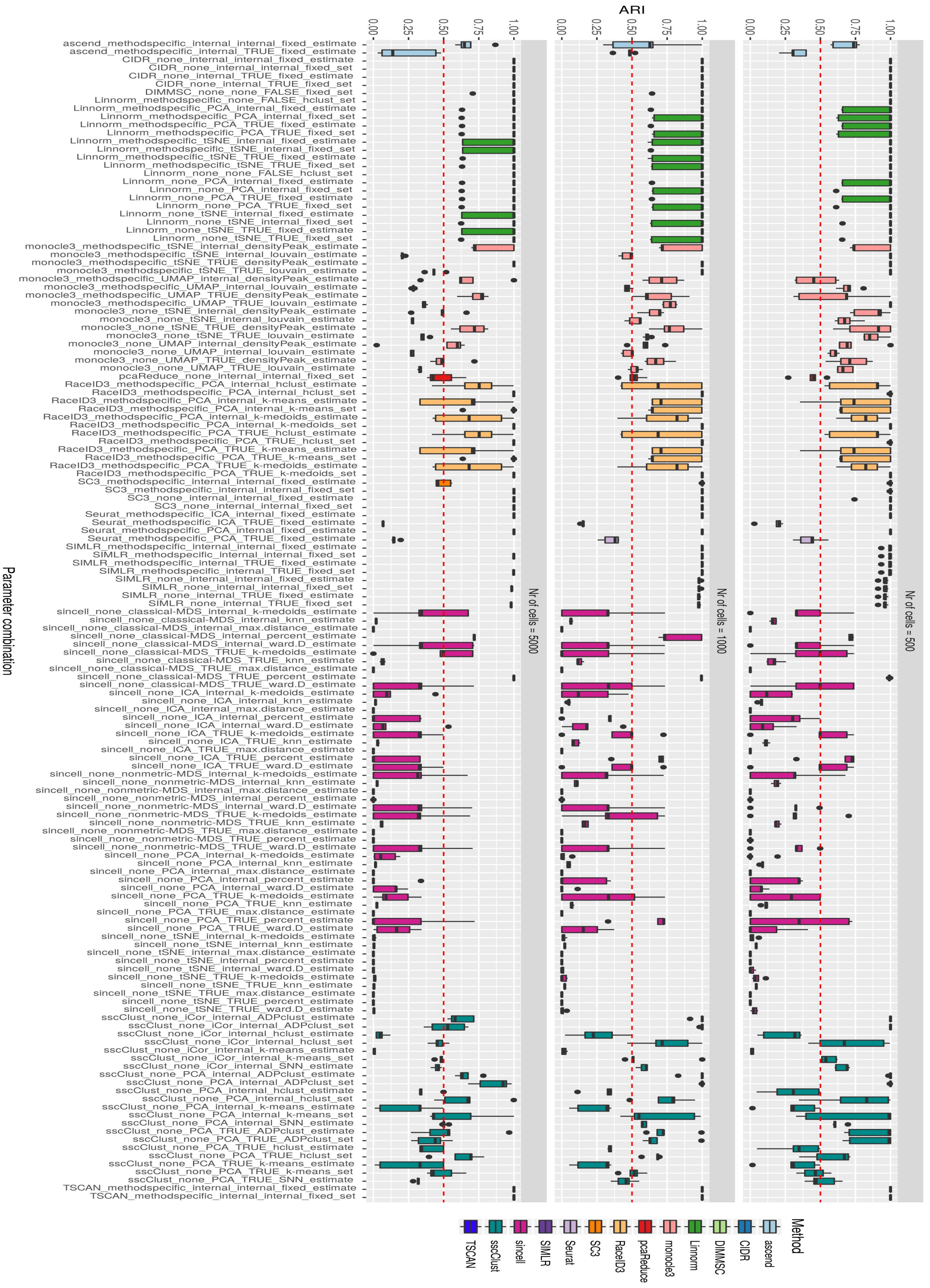

Supplementary Figure 12. **Accuracy of the methods applied to simulated dataset from Setup 1 with 4 cell groups.** Performance of 143 parameter combinations on simulated datasets. Selected results are from the balanced group design across all the runs.

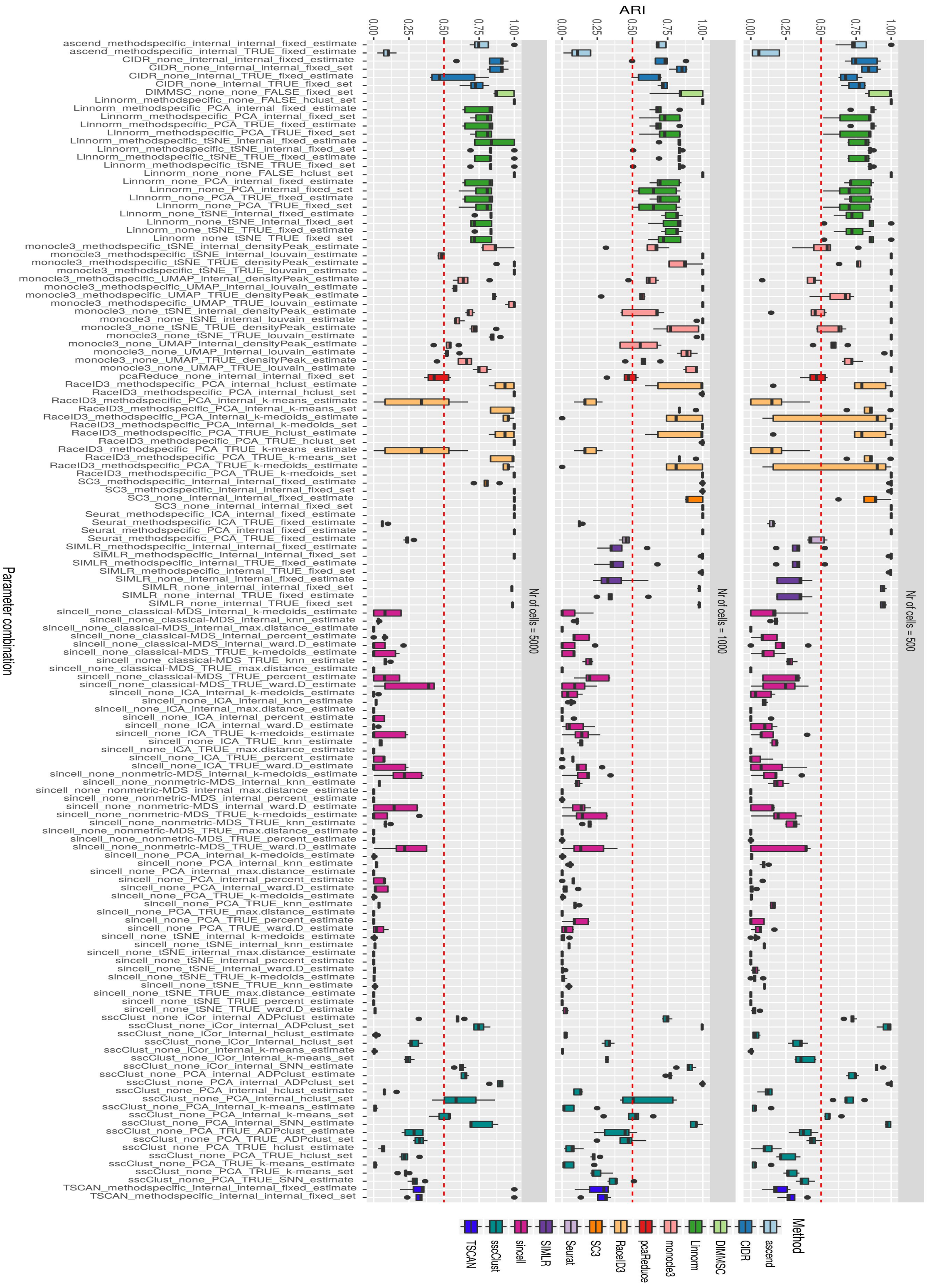

Supplementary Figure 13. **Accuracy of the methods applied to simulated dataset from Setup 1 with 8 cell groups.** Performance of 143 parameter combinations on simulated datasets. Selected results are from the balanced group design across all the runs.

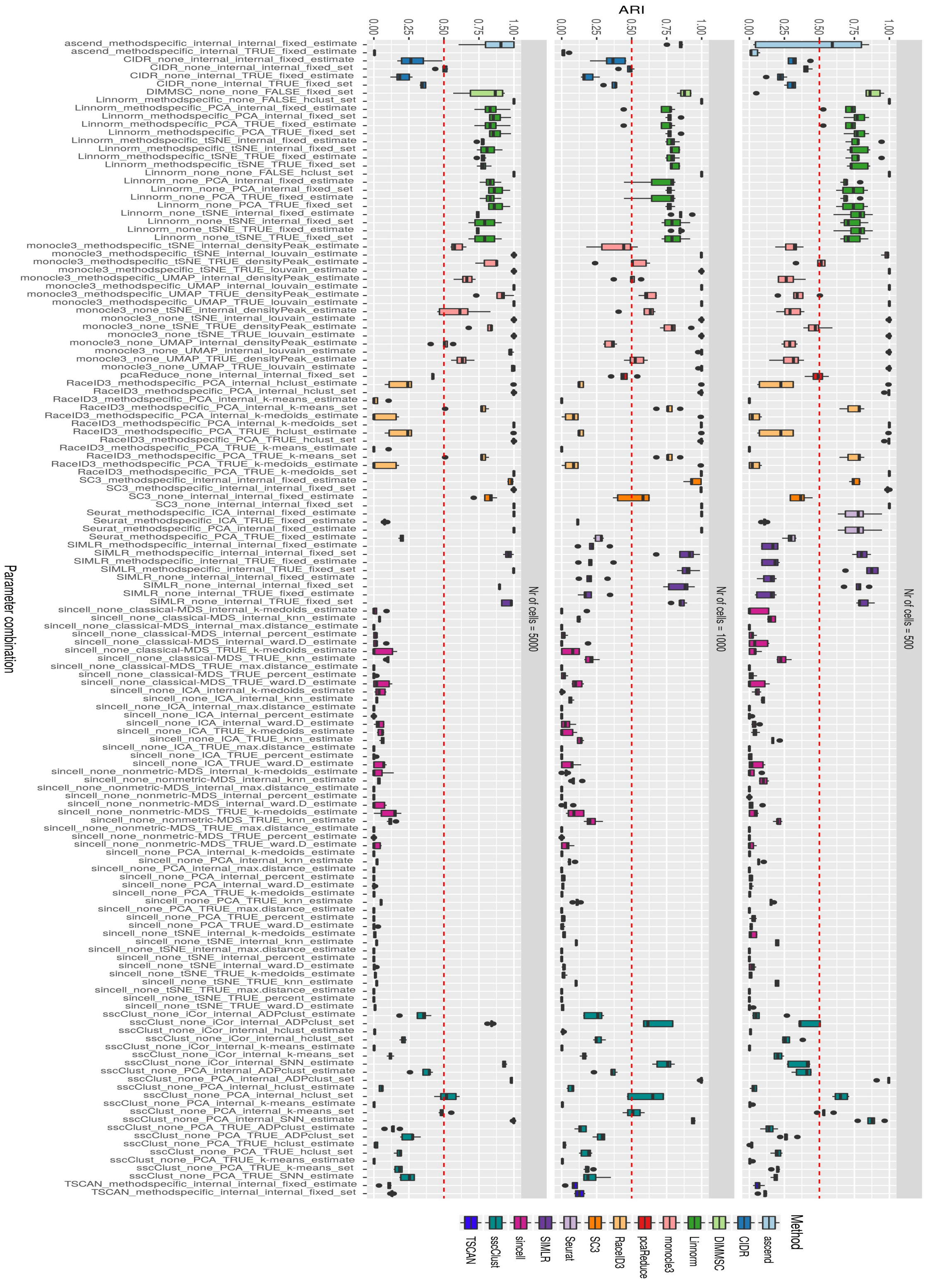

Supplementary Figure 14. Accuracy of the methods applied to simulated dataset from Setup 1 with 16 cell groups. Performance of 143 parameter combinations on simulated datasets. Selected results are from the balanced group design across all the runs.

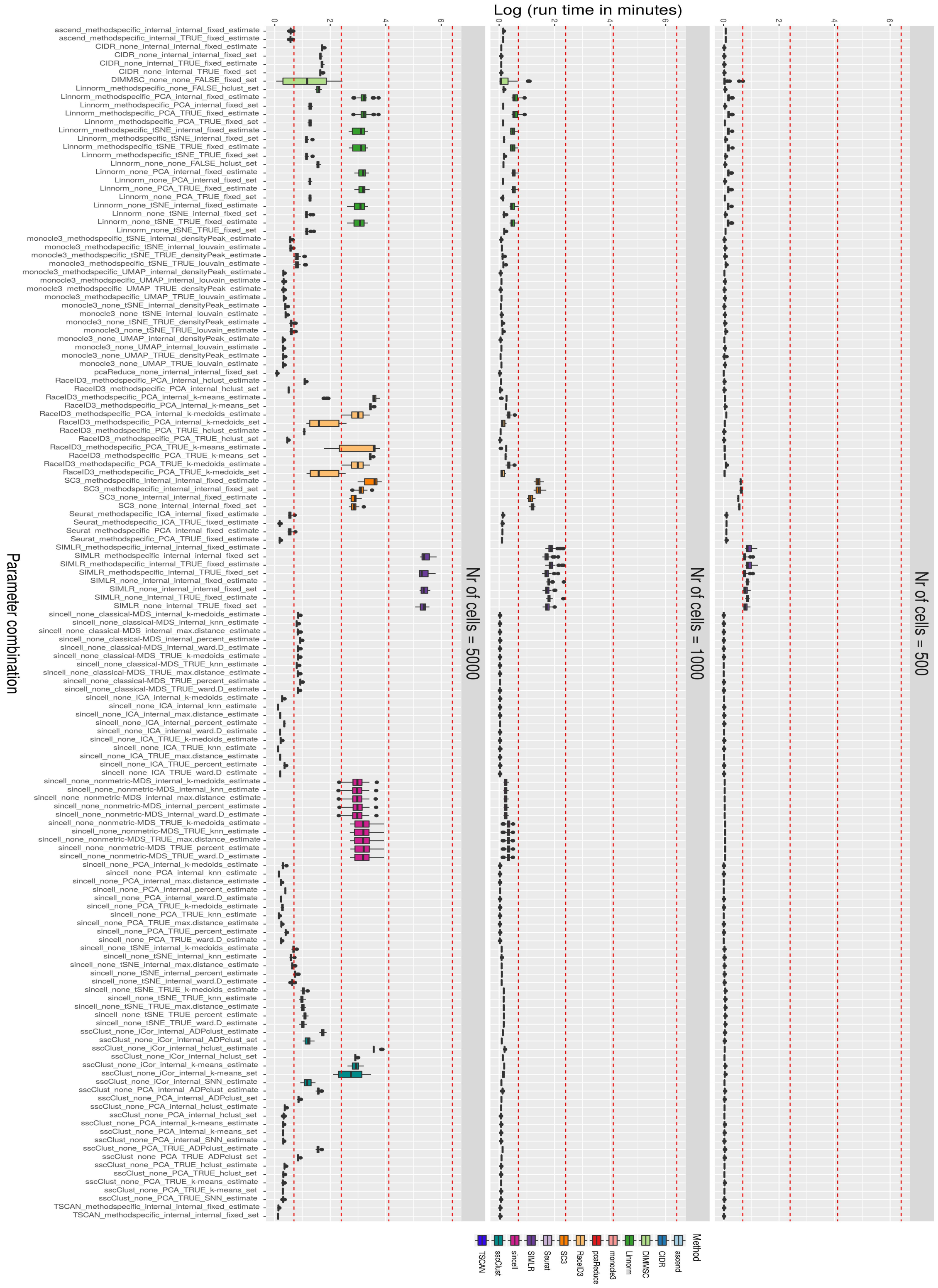

Supplementary Figure 15. Run times for the methods applied to simulated datasets from Setup1. Run times of 143 parameter combinations applied to simulated datasets from Setup 1. Selected results are from the balanced group design across all the runs.
